# Current perspective on production and applications of microbial cellulases: a review

**DOI:** 10.1186/s40643-021-00447-6

**Published:** 2021-10-05

**Authors:** Nisha Bhardwaj, Bikash Kumar, Komal Agrawal, Pradeep Verma

**Affiliations:** 1 Bioprocess and Bioenergy Laboratory, Department of Microbiology, Central University of Rajasthan, NH-8, Bandarsindri, Kishangarh, Ajmer, Rajasthan 305817 India; 2 Department of Chemical Engineering, Institute of Chemical Technology, Nathalal Parekh Marg, Matunga, Mumbai, Maharashtra 400019 India

**Keywords:** Cellulase, Cellulose, Mechanism, Biorefinery, Techno-economic aspects

## Abstract

The potential of cellulolytic enzymes has been widely studied and explored for bioconversion processes and plays a key role in various industrial applications. Cellulase, a key enzyme for cellulose-rich waste feedstock-based biorefinery, has increasing demand in various industries, e.g., paper and pulp, juice clarification, etc. Also, there has been constant progress in developing new strategies to enhance its production, such as the application of waste feedstock as the substrate for the production of individual or enzyme cocktails, process parameters control, and genetic manipulations for enzyme production with enhanced yield, efficiency, and specificity. Further, an insight into immobilization techniques has also been presented for improved reusability of cellulase, a critical factor that controls the cost of the enzyme at an industrial scale. In addition, the review also gives an insight into the status of the significant application of cellulase in the industrial sector, with its techno-economic analysis for future applications. The present review gives a complete overview of current perspectives on the production of microbial cellulases as a promising tool to develop a sustainable and greener concept for industrial applications.

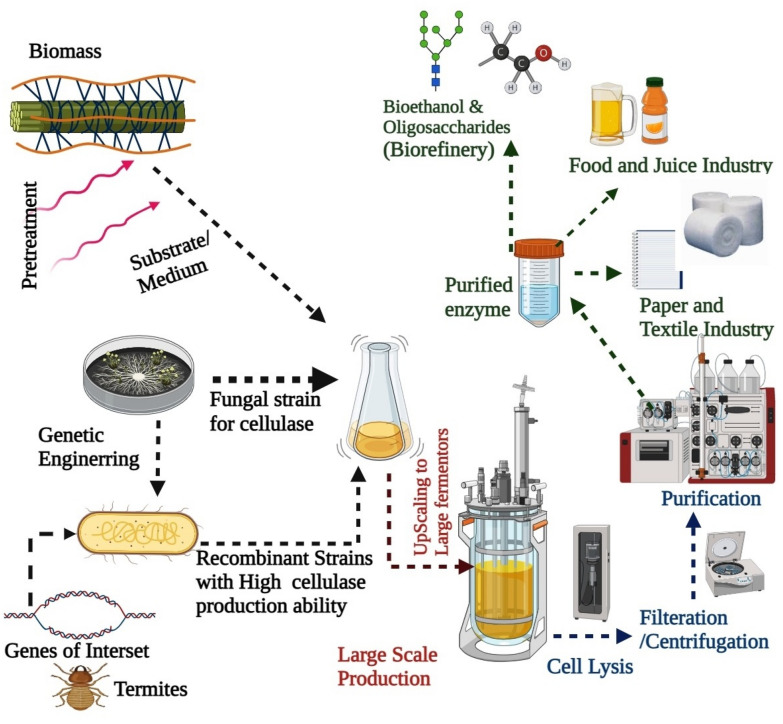

## Introduction

The continuous increase in worldwide industrialization has made researchers find economical ways to fulfill the growing demand. Industries like automobiles, textiles, animal feed, detergent, paper, health care needs, food processing, manure, wine making, and waste management have shown a gradual increase in their demand. Hence, it is necessary to fulfill these requirements without affecting the economy or any harsh effects on the environment. All the industries require various parameters to be considered, such as the use of enzyme-based catalysis, biodegradable and cost-effective raw materials, and low risk of environmental effects (Ding et al. 2008).

Lignocellulosic biomass (LCB) obtained from agricultural and forestry industries can serve as suitable raw materials as they are abundantly available throughout the year (Hou et al. 2020; Pandey and Negi 2020; Verma et al. 2018; Zhao et al. 2019). Cellulose is the main polysaccharide of lignocellulosic plant biomass, its hydrolysis to glucose using enzymes comprises the synergy of three enzymes, i.e., endoglucanases (EC 3.2.1.4), exoglucanases or cellobiohydrolases (EC 3.2.1.91), and β-glucosidases (EC 3.2.1.21). The glycosidic bonds present in the cellulosic biomass are hydrolyzed randomly by endoglucanases in the crystalline and amorphous regions (Sharma et al. 2016; Teter et al. 2014), resulting in the oligosaccharides generation. These oligomers are cleaved by exoglucanases in the reducing and non-reducing ends to generate cellobiose which is further hydrolyzed by β-glucosidases to release sugar molecules such as glucose (Allardyce et al. 2010; Juturu and Wu 2014a, b). Although a massive amount of LCB is being used for various applications worldwide, many unused and partially hydrolyzed raw materials due to the high processing cost are gathering in the biosphere, causing pollution is under focus currently. It has been noticed that in most bioprocess-based industries, the cellulose bioconversion process from LCB is affected by microcrystalline structure of the cellulose-rich materials. Although plenty of conventional physical and chemical methods have been utilized to date for the pretreatment of these materials with interesting output, some secondary contamination was also observed, negatively affecting their utility. Therefore, microbial enzymes are a suitable alternative for increasing the effectiveness of cellulosic bioresources (Premalatha et al. 2015).

Since two decades, cellulase has gained enormous attention as an industrially important enzyme with a wide range of applications. Cellulases are utilized for the saccharification of cellulose which is the innermost component of the lignocellulosic plant biomass. This process of saccharification using cellulase results in the release of glucose that can further be converted to biofuel such as bioethanol by using ethanologenic microorganisms (Nguyen et al. 2018; Raj and Krishnan 2018). Similarly, cellulase and pectinase cocktails have shown better fruit juice extraction and clarification in the food processing industries. Recently, cellulase has been considered the 3rd most important industrial enzyme in the retail market worldwide (Oberoi et al. 2010). Hence, considering these facts, cellulase production has been considered as an important step for the economical use of renewable lignocellulose (raw materials) for the generation of value-added products such as ethanol, single-cell proteins, and other chemicals (Bhardwaj et al. 2019; Kumar et al. 2018). The production process of cellulase is quite expensive and contributes to 50% of the total hydrolysis cost. Therefore, lignocellulosic plant biomass can be considered as an economical raw material for cellulase production and other industrial by-products (Verma et al. 2011; Guldhe et al. 2017). The cellulolytic enzymes are primarily obtained from the microbial origin with noticeable enzymatic activity and stability variations, e.g., physical parameters like pH and temperature. Numerous bacteria, protozoans, fungi, animals, and plants have been reported for their ability to produce cellulases.

**Fig. 1:**
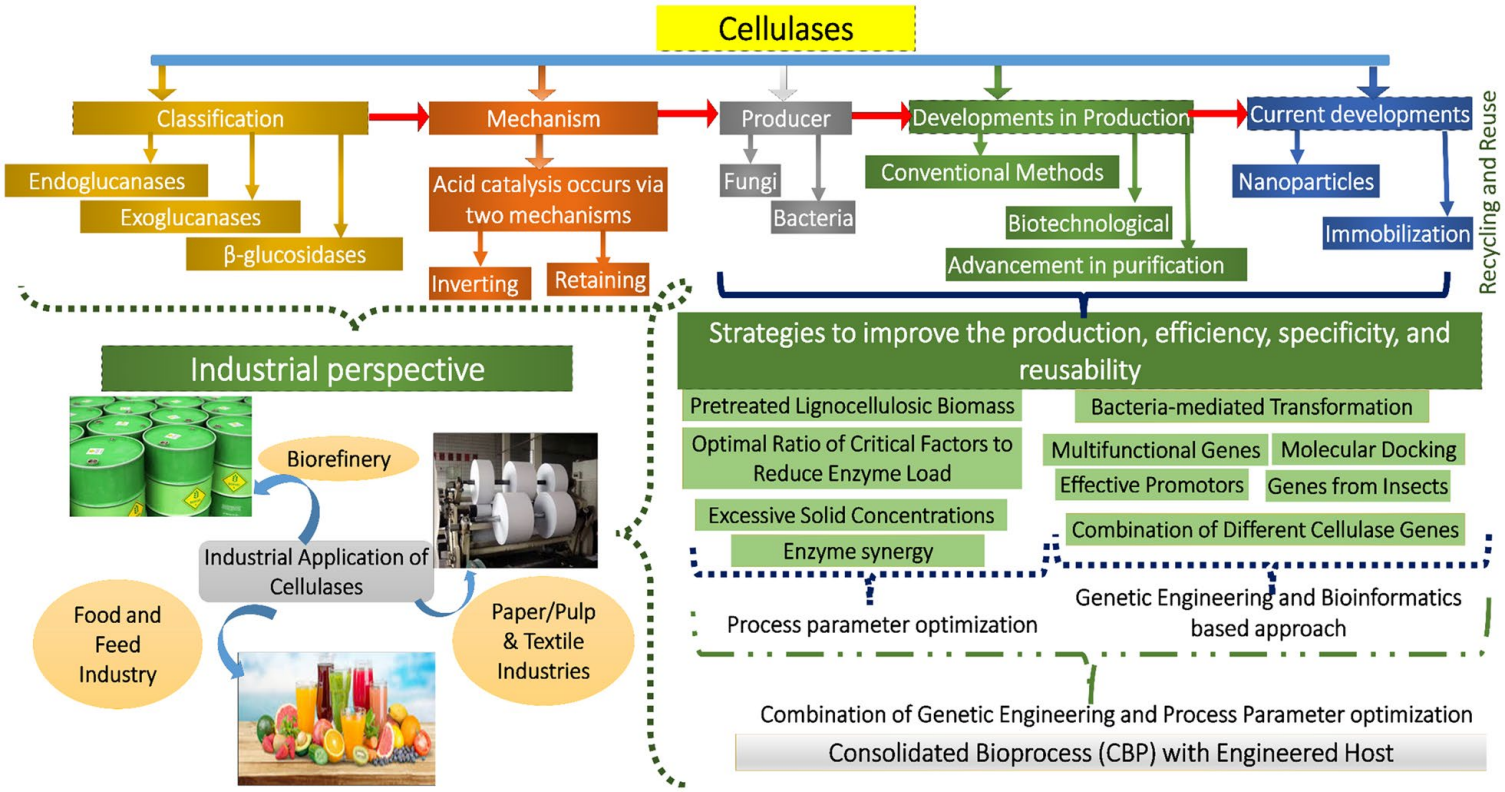
Schematic representation of the various important aspects of microbial cellulase

Hence, with the concern of high demand, the requirement of finding new microbial isolates by exploring different ecological habitats has become essential. The present review includes up-to-date exploration of cellulases, emphasizing the source, mechanism, and methods for enhanced cellulase production with high activity, specificity, and reusability. The review also gives insight into the major application of cellulase in the industrial sector with its techno-economic analysis for future applications (Fig. 1).

## Classification and mechanism of action of cellulolytic enzymes

Cellulolytic enzymes are mainly designated under three groups, first endoglucanases (EC 3.2.1.4) (1,4-β-d-glucan-4-glucanohydrolase or carboxymethyl cellulase), second exoglucanases [Cellobiohydrolase (EC 3.2.1.91) and cellodextrinase (EC 3.2.1.74)] and third β-glucosidases (EC 3.2.1.21) (Hasunuma et al. 2013). All three cellulase groups consist of members in separate GH families based on the CAZy database classification (Lombard et al. 2014). The classification of cellulases is prepared according to the depolymerization stage of the targeting substrate. The glycosidic bonds present in crystalline and amorphous cellulose are randomly hydrolyzed by endoglucanases and lead to the production of oligomers with varying polymerization degrees (Sharma et al. 2016; Teter et al. 2014). However, Szijártó et al. (2008) have also experimentally proved that the EG is more active toward crystalline cellulose, whereas amorphous cellulose is prone to action by CBH. After this β-1,4-glycosidic bonds present at the reducing and non-reducing ends of the oligomers are hydrolyzed by the exoglucanases and produce cellobiose which is degraded further to glucose by β-glucosidases (Hasunuma et al. 2013; Juturu and Wu 2014a, b). Some other critical enzymes that catalyze reversible phosphorolytic cleavage and epimerization are also grouped as part of the cellulase enzyme complex. Cellobiose phosphorylase or cellobiase (orthophosphate α-d-glucosyl transferase, EC 2.4.1.20) catalyzes reversible phosphorolytic cleavage of cellobiose to glucose. Cellodextrin phosphorylase (1,4-β-d-oligoglucan orthophosphate α-d-glucosyl transferase, EC 2.4.1.49) catalyzes the conversion of cellodextrins (cellotriose to cellohexose) to glucose. Cellodextrin phosphorylase does not act on cellobiose. Cellobiose epimerase (EC 5.1.3.11) catalyzes the epimerization of disaccharides like cellobiose into 4-*O*-β-d-glucosylmannose (Sharma et al. 2016).

Additionally, the cellulase enzyme mixture also consists of other accessory proteins such as swollenin and lytic polysaccharide monooxygenase (Harris et al. 2014), facilitating the cellulose degradation. *Trichoderma reesei* protein called the exoproteome is a widely utilized cellulase producer at the industrial level that consists of two cellobiohydrolases (approximately 70% of total proteins), three types of endo-1,4-β-glucanases, one lytic polysaccharide monooxygenase, and one β-glucosidase (Bischof et al. 2016; Herpoël-Gimbert et al. 2008). The cellobiohydrolases are extensively suggested for their necessity in the effective degradation of type I crystalline cellulose (a major allomorph of plants crystalline cellulose Gusakov et al. 2007; Morozova et al. 2010; Szijártó et al. 2008). The enzymatic saccharification rate is reported to be effectively improved when the crystalline cellulose I is disrupted to other crystalline allomorphs (Chundawat et al. 2011; Igarashi et al. 2007; Szijártó et al. 2008).

**Fig. 2:**
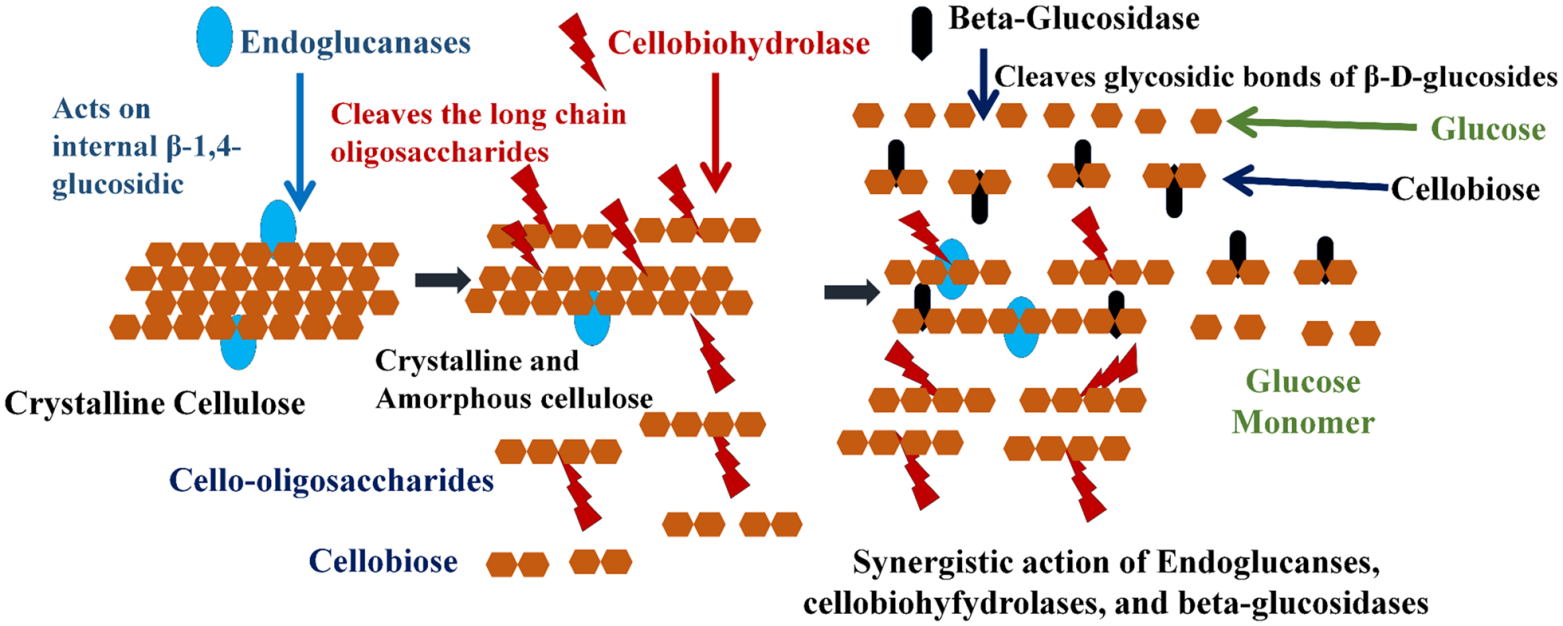
Mechanism of cellulolytic enzymes (adapted with permission from Kumar and Verma 2020a)

The synergistic action of the cellulase enzyme complex controls the bioconversion of cellulose to glucose that can be explained in two steps (Fig. 2). The first step involves the action of exo- and endoglucanases that cause a reduction in the polymerization degree of cellulose in the stage of liquefaction and releases cellobiose. β-Glucosidase is involved in the second step that converts cellobiose into glucose (Maeda et al. 2013).

Similar to hydrolytic enzymes, a water molecule is inserted into the substrate by cellulases during a nucleophilic attack. All the three enzyme types, i.e., endoglucanases, exoglucanases, β-glucosidase shows synergistic action by the similar types of action mechanism known as acid catalysis. The acid catalysis occurs in two different mechanisms, i.e., inverting and retaining. The action pathway of cellulases mainly depends on the distance of the catalytic sites. During inverting mechanism, there is no enzyme–substrates complex formation throughout the reaction, and the hydrolysis process is directly achieved by sequential deprotonation. The carboxylic ends present in the amino acid residues are involved in the retaining mechanism acting as a nucleophilic base that attacks the glycosidic bond, breaks it, binds itself to an oligomeric fraction, and forms an enzyme–substrate complex (Ng and Cheung 2011; Qi 2017).

The cellulase adsorption onto the insoluble cellulose surface is vital for the effective hydrolysis process. The cellulose-binding domain is mainly responsible for adsorption (Linder and Teeri 1997). In fungal cellulases, the cellulose-binding domain mainly consists of 40 amino acids module kept in the CAZy database as carbohydrate-binding module family 1 (CBM 1) (Boraston et al. 2004). The CBM 1 inactivation or removal in *T. reesei* cellobiohydrolase I (CBH I) has dramatically reduced its binding capacity and hydrolysis activity on crystalline cellulose (Amore et al. 2017; Cruys-Bagger et al. 2013; Reinikainen et al. 1992). In contrast, CBM 1 to CBM 1-lacking cellulases fusion can efficiently improve their hydrolysis activities on crystalline cellulose (Szijártó et al. 2008).

The cellulase enzymes structure and substrate binding mechanism vary from microorganism to microorganism (Juturu and Wu 2014a, b). Fungal and aerobic bacterial cellulases are released extracellularly from the cell as free molecules into the medium. On the other hand, in case of anaerobic bacterial cellulases it remains linked to the surface of the cell by forming a protein complex known as cellulosome (Doi and Kosugi 2004). Cellulases from aerobic microorganisms are found in different architecture involving two domains linked by a peptide. One is the cellulose-binding domain, and another acts as a catalytic site (Park et al. 2010). Several other structures with different functions can be found on the protein (Rabinovich et al. 2002).

## Overview of cellulolytic enzymes production

Microorganisms such as bacteria and fungi are good producers of cellulolytic enzymes, although fungi were more suitable for cellulase producers due to their extracellular properties. Thus, continuous exploration in search of new microorganisms (Table 1) has become essential to favors industrial needs (Premalatha et al. 2015). On a commercial scale, several fungal and bacterial strains have been utilized to produce cellulases. Commercially, substrates such as cellulose or carboxymethyl cellulose (CMC) have been used to produce cellulase; however, are very costly. Thus, scientists attempted to replace this costly substrate with naturally occurring cellulose-rich biomass such as agricultural and forest residues (De Almeida et al. 2019; ﻿Crognale et al. 2019﻿;  Goldbeck et al. 2013).

**Table 1:** Cellulases production potential of microbial strains by utilizing plant residues as carbon sources

Microorganisms	Carbon sources	Yield	References
*Aspergillus niger*	Corn stover	6.28 U/mL	Xue et al. (2020)
*Rhizopus oryzae* UC2	Oil palm frond leaves	CMCase 94.68 U/g, FPase 25.46 U/g, β-glucosidase 145.47 U/g	Ezeilo et al. (2019b)
*Trichoderma asperellum* UC1	Raw oil palm frond leaves	CMCase 136.16 IU/g, FPase 26.03 U/g, β-glucosidase 130.09 IU/g, xylanase activity 255.01 U/g	Ezeilo et al. (2019a)
*Trichoderma harzianum*	Rice straw	Cellulase 12.7 U/g, xylanase 110.7 U/g	Syuan et al. (2018)
*Trichoderma harzianum*	Domestic wastewater	FPase 5.4 U/mL, CMCase 8.2 U/mL	Libardi et al. (2017)
*Cellulomonas uda* NCIM 2353	Waste date seeds	175.96 IU/mL	Swathy et al. (2019)
*A. thermoaerophilus*, *B. borstelensis*	Sugarcane bagasse	22.18 and 33.3 IU/mL of endoglucanase	Ejaz et al. (2020)
*Aspergillus japonicus* URM5620	*Passiflora edulis* peel waste	Total cellulase FPase (1.2 U/mL) endoglucanase CMCase 1.7 U/mL	Silva et al. (2019)
*Bacillus velezensis* ASN1	Waste office paper	2.42 U/mL	Nair et al. (2018)
*Trichoderma reesei*	Duckweed (Lemna minor)	6.5 FPU/mL	Li et al. (2019)
*Trichoderma reesei* NCIM 1186, *Penicillium citrinum* NCIM 768	Wheat bran	6.71 FPU/gds	Lodha et al. (2020)

## Utilization of pure cellulose as a substrate for cellulase production

Pure cellulose obtained from microbes and biomass processing can be used as substrates to produce cellulases (Azeredo et al. 2019; Thulluri et al. 2021). Kumar et al. (2018) demonstrated the utilization of commercially available cellulose (CMC) as the substrate for cellulase production using fungal strain *Schizophyllum commune* NAIMCC-F-03379. They compared it with the utilization of different LCB as a substrate. The study showed that wheat bran and CMC showed comparable CMCase and FPase activity. In contrast, all other biomass, i.e., rice straw, rice husk, wheat straw, and sugarcane bagasse, showed poor cellulase activity as compared to commercial cellulose (Kumar et al. 2018). Although cellulase enzyme preparation from fungi is in trend, cellulase-producing bacteria have attracted significant attention due to their strong adaptability (Sadhu et al. 2013a). A total of 10 cellulase-producing bacterial strains were reported from Min pig manure, with the best enzyme producing ability in *Bacillus velezensis* (Li et al. 2020). Gupta and Samant (2012) demonstrated the application of Whatman filter paper rich in cellulose as a substrate for cellulase production utilized by cellulolytic bacteria. The obtained cellulolytic enzyme was then applied for simultaneous saccharification and fermentation (SSF) for efficient ethanol generation ability. Similarly, Li et al. (2009) demonstrated recombinant expression of thermostable cellobiohydrolase gene from *Chaetomium thermophilum*. *Pichia pastoris* efficiently utilized cellulose-rich microcrystalline cellulose for cellulase production. The purified enzyme was found to be thermotolerant (60 °C) and stable at acidic pH (5.0) and showed high saccharification of cellulose substrates such as filter paper.

Although the production of cellulase utilizing LCB as a substrate is lower than that of a pure cellulose-containing culture medium. But the variable structural complexities and low solubility of the pure crystalline cellulose can limit its application as a substrate for cellulase production (Lynd et al. 2002). The commercially available cellulose derivatives are soluble and can result in enhanced cellulase enzyme production in a shorter duration due to the non-requirement of time for overcoming recalcitrance lignin (Chukwuma et al. 2020; Kucharska et al. 2018). But the cost of these derivatives compensates for the time required while using the LCB as substrate. Also, the application LCB helps in the production of other auxiliary enzymes such as hemicellulases, laccases, and LPMOs (Lopes et al. 2018; Obeng et al. 2017). Thus, selecting a suitable biomass/substrate is critical to increasing the potential of enzyme cocktail for saccharification in consolidated bioprocessing in lignocellulosic biorefinery (Østby et al. 2020).

## Utilization of LCB as a substrate for cellulase production

In a study by Marques et al. (2018), a total of fourteen (14) endophytic fungal strains were randomly chosen and prospected for cellulases and xylanases production by solid-state fermentation (SsF). Initially, fungi were cultivated in a mixture (1:1 w/w) of sugarcane bagasse and wheat bran for 7 days at 28 °C. In the initial screening, a total of four (4) fungi, i.e., *Cladosporium cladosporioides* PAJ 03, *Phomopsis stipata* SC 04, *Trichoderma viridae* PAJ 01, and *Botryosphaeria* sp. AM 01 shown endoglucanase activity in a range of 42.79 ± 1.6 U/g to 88.51 ± 1.0 U/g. The other four (4) endophytic fungi, i.e., *Saccharicola* sp. EJC 04, *Paecilomyces* sp. SF 021, *Ustilaginoidea* sp. CV 04, and *Ustilaginoidea* sp. XYA 04 exhibited β-glucosidase activity in a range of 21.72 ± 3.05 U/g to 51.56 ± 2.7 U/g. Among these fungal strains, maximum xylanase and β-xylosidase activity of 694.33 and 4.87 U/g was reported in fungi, *P. stipata* SC 04, and *Botryosphaeria* sp. AM 01, respectively. Further, these eight (8) endophytic fungi were grown in media substituted with lignocellulosic substrates, i.e., 1:1 (w/w) of cotton seed meal and wheat bran. Maximum endoglucanase and β-glucosidase activities of 184.74 ± 6.0 and 92.28 ± 9.57 U/g were observed with *Botryosphaeria* sp. AM01 and *Saccharicola* sp. EJC04, respectively (Marques et al. 2018). Thus, suggesting potential endophytic strains capable of high cellulase enzyme system utilizing LCB as substrate. Similarly, various thermophilic and mesophilic bacterial strains were studied for the production of cellulase and belonged to genera *Cellulosimicrobium*, *Clostridium*, *Thermomonospora*, *Cellulomonas*, *Ruminococcus*, *Erwinia*, *Bacteriodes*, *Bacillus*, *Streptomyces*, *Microbispora*, *Fibrobacter*, *Acetovibrio*, *Paenibacillus*, and *Aspergillus* (Ahmad and Khare 2018; Cai et al. 2019; Mohapatra et al. 2018; Oliveira et al. 2018b; Prajapati et al. 2018; Liang and Xue 2017; Sriariyanun et al. 2016). Commercial fungal strains of *Trichoderma* are a well-known and commonly preferred fungal species for the production of cellulase utilizing cheap and readily available renewable substrates such as spruce, bagasse, wastepaper, dairy manure, and willow (Lan et al. 2013; Wang et al. 2020a).

Additionally, some food processing industries waste rich in lignocellulosic content such as brewery spent grain (BSG) and sugarcane bagasse (SCB) can be used for the production of cellulases using different fungal strains, i.e., *Aspergillus niger* CECT 2700*, A. niger* CECT 2915, and *A. niger* ITV-01 (Moran-Aguilar et al. 2021). The BSG and SBC are first subjected to alkaline/boiling water/autoclave pretreatments then subjected to SsF with selected *Aspergillus niger* strains. The maximum cellulase activity of 6.23 U/g using *A. niger* CECT 2700 and BSG pretreated with boiling water was reported (Moran-Aguilar et al. 2021).

Table 1 represents a summarized list of microorganisms used for cellulase production utilizing various LCB as substrate.

## Cellulase production development strategies at laboratory and industrial scale

Numerous strategies have been employed to increase cost-effective and economically feasible of the cellulolytic enzyme production using sustainable approaches. These include exploring different suitable carbon sources and pretreatment of agro-residues before using them for enzyme production. The media optimization and the role of different media components can be examined. In addition, the assessments of different process parameters have to be examined to increase cellulase production (Verma and Kumar 2020). Further advancement in cellulase production via microbial fermentation processes is also required. Further, microbial co-productions of other essential molecules is required for enhancing the cost efficiency of the overall process. Also, to increase cellulase efficiency via reusability concept and exploitation of different molecular approaches have to be examined for enhancing cellulase activity and efficacy. Some of the strategies have been discussed below.

## Utilization of suitable carbon source and pretreatment necessity of LCB

The utilization of carbon sources can cause more than 50% of the total cost of enzyme production, e.g., pure glucose (Ellilä et al. 2017). The increasing demand for cellulase in various industries has led researchers to find multiple resources that can reduce production costs and be sustainable. As discussed earlier, microbial strains can be used to produce cellulase enzymes utilizing LCB obtained from forestry and agricultural residues as cost-effective raw materials that are abundantly available in nature (Kumar et al. 2008). Lignocellulosic plant residues can be an effective alternative to the costly carbon sources as they are cheap, renewable, abundant, and good nutrients sources for the microorganisms involved in cellulase production (Saini et al. 2017). Many lignocellulosic residues, such as wheat bran, sugarcane bagasse, rice straw, wheat straw, grape stalk, seeds, fruit pomace, corn cob, and soy bran, have been evaluated in the production of cellulases (Jampala et al. 2017; Masutti et al. 2015). Further, variation in atmospheric conditions results in different plant diversity and compositional structure, thus causing variations in the available waste. In Brazil, sugarcane bagasse is an effective alternative to the expensive carbon sources due to its large availability at the sugarcane mills (Vasconcellos et al. 2015).

Additionally, the substrates used in the production process acts as an enzyme inducer, and their source microorganism may produce enzymatic cocktails with diverse catalytic abilities for cellulose breakdown (Li et al. 2017a; Pandey et al. 2016). Similarly, lignocellulosic substrates can be used as a suitable alternative to commercial inducers by producing a number of enzymes leading to better cellulose hydrolysis (Cunha et al. 2012). The use of waste biomass for cellulase production could reduce the production cost and partly address the environmental LCB disposal problems (Gomes et al. 2015).

Although celluloses are available in considerable quantities in the LCB, their accessibility to microbes is poor due to their natural recalcitrance properties in biomass. Thus, before using LCB as the substrate for cellulase production, it can be subjected to pretreatment to improve cellulose accessibility to microorganisms (Saini et al. 2017). After optimization and a suitable fermentation process, pretreatment of biomass can enhance the production of enzymes. The pretreatment of biomass is an effective method to disrupt the LCB complex structure, which increases the cellulose accessibility for fungal attack (Rodríguez-Zúñiga et al. 2014). Even though different pretreatment methods are known, such as biological, physical, chemical, and physicochemical pretreatment and their use mainly depends on the physio-chemical characteristics of the raw materials. Although various pretreatment methods are available, the two methods, i.e., chemical and thermochemical pretreatment, are currently in demand for most industrial applications (Alvira et al. 2010).

The major limitation associated with pretreatment methods is the generation of inhibitors, such as furfural and 5-hydroxymethylfurfural, etc. (Scordia et al. 2013). Thus, the type of pretreatment method strongly affects the proper growth of fungi as the presence of fermentation inhibitors compounds can inhibit fungal growth. Several types of research have been focused on minimizing these inhibition problems (Almeida et al. 2007). Vasconcellos et al. (2015) reported an improved enzyme production by removing the phenolic compounds, which are the known potential inhibitors of cellulase production. Sonication-based treatment of fermentation broth is suggested to have improved cellulase, xylanase, and pectinase enzymes production using different microorganisms (Delgado-Povedano and De Castro 2015; Jalal and Leong 2018). Increased glucose production from *Bacillus licheniformis* α-amylase has been reported using ultrasound treatment at 100% amplitude for 1 min the ultrasound treatment to the sorghum grain slurry (Shewale and Pandit 2009). The amylase enzyme obtained by following this method has enhanced amyloglucosidase enzyme saccharification (by 8%).

## Media optimization and process parameters using statistical tools for enhanced cellulase production

The various optimization studies have been performed to increase cellulase production using one factor at a time (OFAT) and different statistical approaches (Bhardwaj et al. 2017). The study shows that optimizing the medium components and the physicochemical process involved in the cellulase production process improved enzyme yield (Shajahan et al. 2017). A well-known statistical tool, Response Surface Methodology (RSM), was studied to optimize the cellulase production process to evaluate the interactions of independent physicochemical parameters in *A. aneurinilyticus* strain BKT-9 and *Schizophyllum commune* COC, (Kumar et al. 2018; Srinubabu et al. 2007). RSM is a multivariate approach with many benefits, such as a smaller number of investigational runs, increased justification of the statistical potentials, and individual and interactive properties of the involved parameters. The Central Composite Design (CCD) is a known statistical design under RSM with a rotatable feature while facing difficulty in star points extension beyond the upper and lower limits designed for each factor in the experimental region. The nutritional and environmental factors of cellulase production were optimized for bacteria obtained from Dal Lake, urban freshwater Himalayan Lake (Srinubabu et al. 2007). Shah et al. (2021) reported a new cellulase-producing strain *Bacillus licheniformis* KY962963, an epiphytic bacteria of marine algae *Chlorococcum* sp. The optimization of nutritional and ecological factors for enhanced cellulase production using *Bacillus licheniformis* KY962963 was performed using the concurrent application of “Plackett–Burman” design and one factor at a time approach. The study suggested that moisture content (75%), K_2_HPO_4_ concentration (2 g/L) and incubation temperature (35 °C), and an incubation time of 3 days were optimum condition for enhanced cellulase production.

## Improvements in cellulase production via microbial fermentation processes

The SsF and solid submerged fermentation (SmF) are the two main methods utilized for the production of cellulase and other enzymes like proteases, xylanase, and pectinases using different microorganisms (Mrudula and Murugammal 2011; Pant et al. 2015). Lignocellulosic feedstocks can be utilized as substrates that act as an inducer for enzyme production during fermentation. SmF possesses several advantages in process control as abundant water reduces the oxygen, temperature, and nutrient concentration gradients, and enzyme recovery is easy in SmF. Additionally, the submerged fermentation process can serve as a well-established technology to develop the fermentation processes for industrial-level production capability (Colla et al. 2016; Hansen et al. 2015).

Similarly, during SsF, an organic substrate is degraded aerobically in the absence of free water to produce the desired end-product. The optimal parameters vary extensively based on the fermentation process. Also, end-product varieties can be generated by using the same substrate in varying operational conditions or by incubating with different microbial strains (de Castro and Sato 2015). Several reports suggest that SsF is an economical method for producing different industrially important enzymes and several other biochemicals by using lignocellulosic feedstock as substrate (Uncu and Cekmecelioglu 2011). There are several reports for cost-efficient cellulase production using SsF with LCB as substrate (Dhillon et al. 2012). It required simple equipment with lower energy needs resulting in high enzyme yield and significantly less processing costs. SsF and SmF using LCB as substrates is an added advantage to the process based on economic concern and promoting the search for effective substrates. Therefore, different strategies have been employed to reduce an enzyme’s production cost, such as SsF using lignocellulosic plant residues as carbon source and enzyme inducer.

## Microbial co-production of other essential enzymes for the overall economy of the processes

Along with cellulase, various other enzymes such as pectinase and xylanase have several applications in different fields such as waste treatment, value-added chemical production, field biofuel generation, etc. (Ali et al. 2013). Therefore, instead of using a single microorganism, researchers focus on those microbial cultures that can produce multiple enzymes (Li et al. 2018b). The biological processes have been used as an effective method for producing these essential enzymes from abundantly available substrates, and the cheap fermentation process is always in high demand (Ravindran and Jaiswal 2016; Singh et al. 2016). Among the applications of pectinase, cellulase, and xylanase co-production help in one critical application: finishing and degumming natural fibers, e.g., ramie fiber. Ramie fiber is composed of hemicellulose and pectin, which requires degumming to fulfill the textile industry’s textile requirements (Zheng et al. 2001). The microbial co-production of essential enzymes (hemicellulase and pectinase) can be advantageous for this specific degumming process.

LCB is rich in lignin, cellulose, and xylan constituents and also several decomposer fungal strains are capable of producing these enzymes simultaneously for their natural growth. Thus, concurrent production of lignocellulolytic enzymes is a suggested method to decrease the overall cost of lignocellulolytic biorefinery. Jampala et al. (2017) suggested concomitant production of cellulase and xylanase from *Trichoderma reesei* NCIM 1186 and its enhancement via using desirability-based multi-objective optimization method.

Ultrasound-assisted treatment of fermentation broth has a positive effect on the co-production of fibrinolytic, cellulolytic, hemicellulolytic, and pectinolytic enzymes using different microorganisms (Avhad and Rathod 2015; Delgado-Povedano and De Castro 2015; Jalal and Leong 2018). Avhad and Rathod (2015) demonstrated that involving ultrasonication to induce enzyme production from microorganisms increased fibrinolytic enzyme yield. The process involved 12 h of bacterial growth from *Bacillus sphaericus* MTCC 3672 with optimized ultrasound treatment parameters of 25 kHz ultrasound irradiation frequency with 40% duty cycle and 160 W power, which enhanced 1.48-fold cellulase production in a 1-L bioreactor (Avhad and Rathod 2015). Similarly, Yadav et al. (2020) demonstrated the effect of the ultrasonic treatment in enhancing the fermentative co-production of cellulase, xylanase, and pectinase enzymes from *Bacillus subtilis* ABDR01. Sonication of fermentation broth at ultrasound power of 90 W using 25 kHz frequency with 70% duty cycle for 5 min gave the maximum cellulase, xylanase, and pectinase production of 22.17 U/mL, and 137.95 U/mL, and 87.82 U/mL, respectively, at the short bacterial growth phase of only 6 h. This study also suggested that the application of ultrasonic irradiation of fermentation broth causes cell cluster disaggregation and enhances the nutrient uptake along with maintaining the cellular integrity of microorganisms. This process caused a remarkable increase in the biomass concentration and end-product of the fermentation process.

## Strategies to increase cellulase efficiency and reusability

Various strategies have been employed to increase cellulase efficiency and reusability. Some of these strategies have been discussed using different molecular approaches, as mentioned below.

## Molecular approaches via recombinant DNA technology to increase cellulase activity and efficacy

**Fig. 3:**
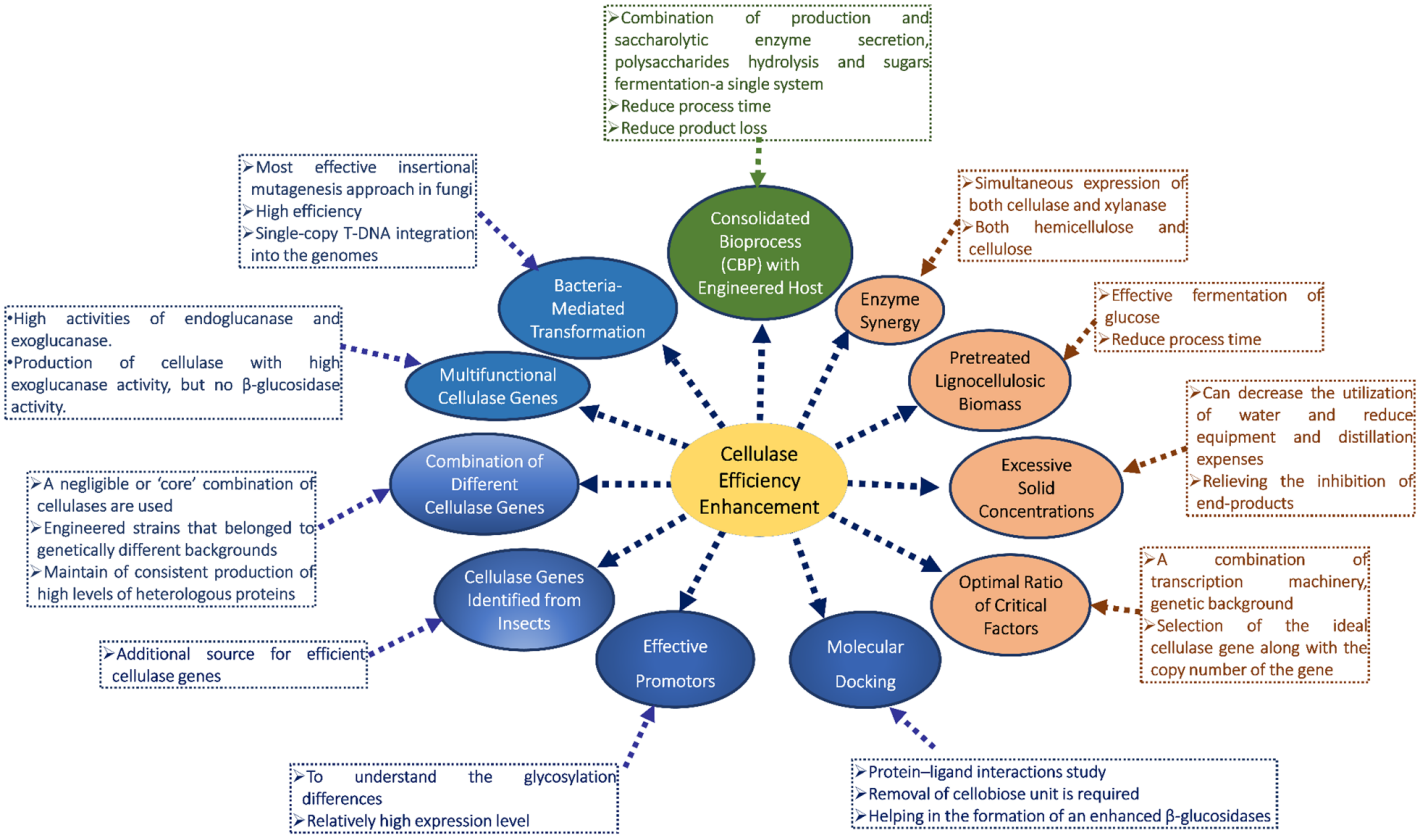
Strategies to increase cellulase efficiency and characteristics

The continuous increase in the requirement of resources for renewable energy has gained attention, and lignocellulosic plant biomass can be considered the most abundantly available carbon source in nature. The enzymatic hydrolysis process of cellulose components served as an effective process for the bioconversion of LCB to bioethanol due to its effective and economic properties (Sukumaran et al. 2009). Although the high production cost of cellulase contributes to half of the total cost of saccharification, it remains one of the major hurdles in the use of lignocellulose as a bioethanol source (Ariffin et al. 2008). Hence, for further reduction in the cost of cellulose-based ethanol production, microbial strains improvement can serve as a potential method for cellulase overproduction. A filamentous fungus *Trichoderma reesei *(Teleomorph *Hypocrea jecorina*) is the most widely studied organism for the complete set of cellulolytic enzymes production. Subsequently, its cellulolytic potential was documented in the late 1960s, and several studies have been performed for developing the mutants capable of producing efficient cellulases using conventional mutagenesis with physical and/or chemical mutagens (Chand et al. 2005; Zhang et al. 2006). Many high cellulase-producing *T. reesei* mutants have been reported, which are currently being used on a commercial scale. Their genetic basis responsible for enhanced production of cellulases is not known properly (Fujii et al. 2010; Zhang et al. 2006). A widely used mutant strain RUT C30 was observed lacking a genomic fragment of 85 kb and missing ~ 30 genes that are involved in various biological processes while using genome walking in combination with complex oligonucleotides for determining the loci deletion (Seidl et al. 2008). The cloning of mutated genes using physical and chemical mutagens is a tedious process, hence, a reason for slow progress in mutations characterization at the molecular level (Gehring et al. 2000). Hence, using practical insertional mutagenesis approaches for discovering gene function in terms of cellulase formation can improve the strain properties and help understand the mechanisms responsible for the high cellulase production. The insertional mutagenesis has advantages, such as the mutated genes are tagged with inserted elements that can be used to identify the flanking sequences and the disrupted genes (Jeong et al. 2002). A flow diagram of strategies employed for increasing the cellulase efficiency and their characteristics are represented in Fig. 3.

## Application of bacteria-mediated transformation

One of the most effective insertional mutagenesis approaches in fungi is *Agrobacterium*-mediated transformation (AMT) because of its high efficiency and mainly leads to single-copy T-DNA integration into the genomes (Michielse et al. 2005; Zhong et al. 2011). A PCR-based method, e.g., TAIL-PCR, can be used to amplify the genomic flanking sequences of T-DNA insertions and can be considered a strong tool for consequent reverse genetic mutants analysis (Combier et al. 2003). Several experiments performed using filamentous fungi in past years have positioned T-DNA as the favored insertion mutagen for the generation of large-scale mutant libraries to identify the mutants of interest and interpret gene functions (Jeon et al. 2007).

Previously, an efficient *Agrobacterium*-mediated transformation (AMT) technique for random insertional mutagenesis has been reported in *T. reesei*, and the T-DNA inserts flanking genomic DNA sequences were released by the use of TAIL-PCR (Zhong et al. 2007). A T-DNA-tagged mutant library formation using AMT was reported for cellulase production improvement in which three putative mutants, i.e., TA-32, TB-87, and TE-6, with increased endoglucanase, cellobiohydrolase, and β-glucosidase activities with improved hydrolysis efficiencies were obtained by using 96-well plate screening on cellulose substrates for rapid growth and plate-clearing zone assay that resulted in 38%, 51%, and 31% increased cellulase activity, respectively, as compared to parental strain QM9414. Additionally, T-DNA was integrated at a single site in the TA-32 genomes, and TE-6 was inserted at two copies into the TB-87 genome. The sequences flanking the T-DNA insertion sites were rescued that explained its huge importance in cellulase production improvement and identification of tagged genes with cellulolytic activity (Zhong et al. 2012).

## Application of multi-functional cellulase genes

*Coprinopsis cinerea,* a modulation in the multi-functional cellulase (mfc) gene expression, was reported using glyceraldehyde-3-phosphate dehydrogenase (gpd) promoter fragments from *Flammulina velutipes*, *Agaricus bisporus*, and *Lentinula edodes*. In the culture liquid Cabm44 that used *A. bisporus* 275 bp gpd fragment, a submerged fermentation was performed using banana peels as a substrate for growth has reported the total cellulase activity of 0.26 U/mL, highest activities of endo-β-1,4-glucanase, i.e., 0.48 U/mL, and endo β-1,4-xylanase of 38.10 U/mL. This study reported the twofold increased lignocellulolytic enzymatic activities over wild-type strain (Yang et al. 2011).

An enhanced exo- and endoglucanase activities from *A. niger* cellulase have been reported to increase the cellulose hydrolysis efficiency and an appropriate enzyme composition. The exoglucanase or endoglucanase protein expression strategy was found helpful for *P*. *rhizinflata* cellulase (AF094747) as it showed high activities of endoglucanase and exoglucanase. *P*. *rhizinflata* cellulase depolymerization function was found better than that of *T. reesei* exoglucanase (Liu et al. 2001; Tsai et al. 2003), that is mainly used for the production of cellulase with high exo-glucanase activity, but no β-glucosidase activity as it may hinder the cellulose into glucose conversion (Xue et al. 2017a).

## Combination of different cellulase genes

To get substantial hydrolysis of cellulosic substrates, at least a negligible or ‘core’ combination of cellulases is required to be produced. Some genes combinations, e.g., *Trichoderma reesei* endoglucanase (Tr-EGII), *Talaromyces emersonii* cellobiohydrolase (Te-CBHI), and *Saccharomycopsis fibuligera* β-glucosidase (Sf-BGLI) were expressed in yeast that resulted in partial hydrolysis of lignocellulose (Lambertz et al. 2014; Olson et al. 2012). Additionally, some engineered strains that belonged to genetically different backgrounds have shown the capabilities of a range of cellulolytic enzymes secretion (Dadwal et al. 2020; Davison et al. 2016, 2019a). Based on previous studies, it was observed that extremely high cellulase (20 FPU/g biomass) and β-glucosidase (20 U/g biomass) loadings could decrease the concentrations of glucose significantly (Banerjee et al. 2010), that suggested that a satisfactory balance of cellulase activity is essential.

The stability of the increased expression cassette and copy number are the two important key steps involved in maintaining consistent production of high levels of heterologous proteins in *S. cerevisiae* in the biotechnological industry (Den Haan et al. 2013). It is necessary to understand the copy number effect on protein production ratios and its influence on hydrolysis and fermentation with the development of methods that supports stable, high copy numbers in yeasts, e.g., POT1-mediated delta (δ) integration (Song et al. 2017).

## Application of cellulase genes identified from insects

Several organisms can utilize cellulose-rich biomass as a food source, such as insects. It involves the presence of cellulases, i.e., endoglucanases, exoglycanases, and β-glucosidases acting synergistically on LCB in their gut and digestive tract. In an approach to find new and efficient organisms that can be potential sources for cellulases, understanding the presence of cellulase in the gut and digestive tract of insects becomes a critical aspect. (Kim et al. 2008). Initially, the endogenous cellulase gene was discovered in termites. In insects, the endogenous cellulase genes have been isolated to date from termites (Nakashima et al. 2002), cockroaches (Lo et al. 2000), and beetles (Sugimura et al. 2003). Endogenous cellulases from insects have been reported in three glycosyl hydrolase families (GHFs): GHF 9 (termites and cockroaches), GHF 45 (beetles), and GHF 5 (beetles) that are phylogenetically and structurally unrelated. Among these, GHF 9 has been studied widely in termites. Based on previous studies, it can be observed that endosymbiotic and termite-derived cellulases are both present in termites (Nakashima et al. 2002; Scharf et al. 2003). An endogenous GHF 9 cellulase expressed in a wood-feeding termite *Coptotermes formosanus* is mainly found in the midgut, and the salivary glands have two independents cellulose-digesting systems. In first digestion of cellulose was carried out by endogenous cellulases that are in the midgut and second in the hindgut, where cellulases from the symbiotic flagellates were utilized (Nakashima et al. 2002). Another report on one endogenous GHF 9 cellulase from termite *Reticulitermes flavipes*, completely expressed in the foregut and the salivary gland, in which three symbiotic cellulases were exceedingly expressed in the hindgut, describing that endogenous and symbiotic cellulases acts in collaboration and serially throughout the whole digestive tract of *R. flavipes* (Zhou et al. 2007). Therefore, it can be concluded that EGs of GHF 9 is limited to the salivary glands, midgut, and foregut of termites, while EGs of GHF 7, GHF 5 and GHF 45 are restricted to the hindgut in which several cellulolytic flagellates are hidden (Ohtoko et al. 2000; Tokuda et al. 2007). In a previous work (Tokuda et al. 2007), researchers reported that termites cellulolytic systems support a dual cellulose-digesting system (Nakashima et al. 2002) instead of a single unified cellulose digestion system (Zhou et al. 2007). A novel endogenous β-1,4-endoglucanase (EG) gene that belonged to the glycosyl hydrolase family 9 (GHF 9) was distributed in the digestive tract of cricket (*Teleogryllus emma*), and it was cloned and characterized. TeEG-I gene consisted of eight exons encoding 453 amino acid residues and existed as a single copy in the *T. emma* genome. TeEG-I holds all properties of GHF 9 members involving catalytic domains and signature motifs that was highly identical with *Mastotermes darwiniensis* (termite) with 64% identical protein sequence, and *Panesthia cribrata* (cockroach) with 62% identical sequence from GHF 9 cellulases. At pH 5.0 and temperature 40 °C from a recombinant TeEG-I, expressed as a 47-kDa polypeptide in *baculovirus*-infected insect Sf9 cells was reported, with 5.4 mg/mL and 3118.4 U/mg of *K*_m_ and *V*_max_ values, respectively, for the CMC digestion. The presence of TeEG-I was observed throughout the digestive tract in Northern and Western blot analysis. This suggested the correlation in the distribution of TeEG-I and cellulase activity in the digestive tract that was observed by immunofluorescence staining and enzyme activity assay, respectively. It also suggested TeEG-I distribution in the entire digestive tract of *T. emma*, which represents functional role of endogenous TeEG-I in the process of cellulose digestion (Kim et al. 2008).

Studies also showed recombinant cellulases from insects and performed significantly better than other cellulases (Hirayama et al. 2010; Kim et al. 2008; Willis et al. 2017). Hirayama et al (2010) reported successful cloning of two termite endogenous β-1,4-endoglucanases gene, i.e., RsEG (salivary gland of *Reticulitermes speratus*) and NtEG (midgut of *Nasutitermes takasagoensis*) gene using *Aspergillus oryzae* as host. The study clearly indicated that these two recombinant endoglucanases are highly efficient than those obtained previously from fungal or bacterial system. Recently, Wilis et al. (2017) performed successful expression of cellulase gene (TcEG1) gene from *Tribolium castaneum* in transgenic switchgrass. This cellulase was active at alkaline pH and helped in auto-hydrolysis of biomass with increased cellobiose release. Thus, this infused ability in biofuel crops to produce its own cell wall-digesting cellulase enzymes would help reduce the costs of cellulosic biofuel production.

## Current promotor utilization

A multi-functional enzyme called EGX, directly isolated from *A. crossean* (an animal) with exo-b-1,4-glucanase, endo-b-1,4-glucanase, and endo-b-1,4-xylanase activities, was reported as the first multi-functional cellulase. Although the enzyme yield was produced from *A. crossean* it was not found optimal for the industrial level application (Wang et al. 2003). Therefore, EGX gene was overexpressed in *Pichia pastoris* and *Saccharomyces cerevisiae* (heterologous ascomycetous hosts) (Gao et al. 2007). Although this study showed that the heterologous expressions of the EGX gene in yeasts are not that suitable for biotechnological applications because of the glycosylation differences and relatively low expression level. Hence, a different strategy was used to overexpress the EGX gene in basidiomycetes that is the use of highly efficient promoters. Another report using this strategy using the controlled *Lentinula edodes* gpd promoter was with the antifreeze protein gene (afp) overexpression which was isolated from *Choristoneura funiferana* (an insect) in basidiomycete *Volvariella volvacea* (Wang et al. 2008).

Similarly, a green fluorescent protein gene (gfp) was overexpressed in basidiomycete *Pleurotus nebrodensis* (Lin et al. 2008). To date, no systematic evaluation of the efficiency of a promotor and heterologous expression of cellulase in basidiomycetes is available. *Coprinus cinereus* was found different from the other basidiomycetes. It is simple to be handled in DNA transformation with the transformation rates of up to 1000 transformants per microgram of DNA through the production of oidium (Granado et al. 1997). Recent studies on the use of molecular approaches in enhancing cellulolytic properties are listed in Table 2.

**Table 2:** Molecular approaches towards enhanced enzymatic properties of cellulases

Microorganism	Gene	Characteristics of recombinant cellulase	References
*Batocera horsfieldi*	Bh-EGase I	SA: 1030.87 IU/mg, 40 °C and pH 4.0	Mei et al. (2016); Zhong et al. (2012)
*Corbicula japonica*	Glycoside hydrolase family (GHF) 9 β-1,4-glucanase gene, CjCel9A	GHF45 (CjCel45A, CjCel45B). Both genes encode ORF of 627 bp corresponding to 208 amino acids Key role as cellulose decomposer in estuarine environments	Sakamoto and Toyohara (2009)
*Bacillus subtilis*	EG gene	Introduced those benefits of commercial bioconversion of complex lignocellulosic materials to biofuels	Ghadiri et al. (2020)
*Streptomyces* sp., *Thermobispora bispora*,* Cellulomonas fimi*, *Streptomyces halstedii*, *Clostridium thermocellum*		Comparative sequence alignment, molecular modeling, and docking analyses were performed taking 5 microbial (3 bacteria and 2 fungi) cellulase enzymes Cellulose-binding site in endoglucanase E of *Clostridium thermocellum* Ser131, Met263, Gln298, and His310 which are reported to have differed with Ala119, Ala255, Ser298, and Cys304 in *Streptococcus* sp. Molecular modeling and docking studies revealed that different microbial cellulase enzymes have the potential towards the use of cellulose as a substrate for the high yield of bioethanol	Paul et al. (2020)
*Talaromyces emersonii*	Cellulase gene (cbh2)	*cbh2* has an open reading frame of 1377 bp, which encodes a putative polypeptide of 459 amino acids Expression of the *T. emersonii cbh2* gene induced by cellulose, xylan, xylose, gentiobiose, and repressed by glucose	Murray et al. (2003)
Fosmid library of Buffalo rumen metagenomic DNA	Acid-cellulase gene (Cel-1)	ORF encoding cellulase consisted of 1176-bp, corresponding to a protein of 391 amino acid and has catalytic domain belonging to glycosyl hydrolase family 5	Dadheech et al. (2018)
*Trichoderma reesei* Rut-C30	*egl1 at the ace1 locus*	Cellulase production by *T. reesei* QS305 performed in the 5-L fermentor, cellulases activity of 10.7 FPU/mL achieved at 108 h, 75.4% higher than that produced by *T. reesei* Rut-C30	Meng et al. (2018)
*Aspergillus niger*		PCR product was 1311 bp, xylanase activity of the reconstructed *A*. *niger* was 3.28 U/mL. The activity of the exoglucanase from the constructed strain was increased to 1.95 U/mL from 0.21 U/mL of the original *A*. *niger*. Reconstructed *A*. *niger* cellulase could hydrolyze wheat straw to release 19.7 g/L of glucose, compared to 12.01 g/L of that released by the cellulase from the original *A*. *niger*	Xue et al. (2019)
*Acinetobacter* sp.		These docking studies revealed that cellulase has the greater potential towards the cellotetraose as a substrate for the high yield of ethanol	Selvam et al. (2017)
Cellulase produced by bacteria in gayal rumen	Umcel-1	Putative gene *Umcel-1* product belonged to the glycosyl hydrolase family 5 and showed the highest homology to the cellulase (GenBank accession no. YP_004310852.1) from *Clostridium lentocellum* DSM 5427, with 44% identity and 62% similarity. Open reading frame of 942 base pairs that encoded a product of 313 amino acids The activity of purified recombinant Umcel-1 was assessed, and the results revealed that it hydrolyzed carboxymethyl cellulose with optimal activity at pH 5.5 and 45 °C	LI et al. (2016a)
*Aspergillus niger*		Exoglucanase increased from the original 0.21 U/mL to 0.89 U/mL of the transformant. Endoglucanase increased from the original 4.51 U/mL to 15.12 U/mL of the transformant. FPA increased nearly 7.1-fold from 0.63 to 4.47 U/mL Cellulase from the transformant retained the halo-stable ability	Xue et al. (2017b)
*Trichoderma reesei cellulase*	(TrCBH2)	The final titer of more than 18 g/L of secreted protein was produced under controlled conditions in small-scale bioreactor cultivations after 60–70 h. High concentration of secreted enzyme in *P. pastoris*	Mellitzer et al. (2014)
*Trichoderma reesei*	Beta-glucosidase gene from *Penicillium decumbens*	The beta-glucosidase activity of the enzyme complexes from two selected transformants increased six- to eightfold and their filter paper activity (FPAs) enhanced by 30% on average	Ma et al. (2011)
*Coprinus cinereus*	Cellulase gene mfc	The highest activities of endo-β-1,4-glucanase (34.234 U/mL) and endo-β-1,4-xylanase (263.695 U/mL) were reached by the *L. edodes gpd* promoter	Cheng et al. (2009)
*C. shiraiana*	CscelA	*CsCelA* expressed in *P. pastoris* high pH stability and affinity. The active protein of 55.3 kDa recombinant cellulase (CsCelA) was 17.44 U/mL and 135 U/g for freeze-dried powder The *K*_max_ and *V*_max_ of CsCelA for sodium carboxymethylcellulose (CMC) were 4.6 mg/mL and 107.2 U/mg, respectively A stable activity from pH 4.0 to 9.0, and at temperatures ranging from 30 to 55 °C	Lü et al. (2015)
*Teleogryllus emma*	Novel endogenous β-1,4-endoglucanase (EG) gene belonging to the glycosyl hydrolase family 9 (GHF 9) TeEG-I	Signature motifs and catalytic domains, of GHF 9 members, sharing high levels of identity with the termite, *Mastotermes darwiniensis* (64% protein sequence identity), and the cockroach, *Panesthia cribrata* (62%), GHF 9 cellulases 47-kDa, Optimal pH and temperature of pH 5.0 and 40 °C *K*_m_ and *V*_max_ values for digestion of carboxymethyl cellulose 5.4 mg/mL and 3118.4 U/mg results indicate that TeEG-I distributed throughout the entire digestive tract of *T. emma*, suggesting a functional role of endogenous TeEG-I in a sequential cellulose digestion process *T. emma* digestion tract	Kim et al. (2008)
*Saccharomyces cerevisiae*	PSE1 and SOD1	*cel6A* of *Neocallimastix patriciarum*, β-glucosidase encoded *cel3A* of *Saccharomycopsis fibuligera* resulted in heterologous protein activity ranging between 10 and 373% high compared to the parental strains grown in complex media	Kroukamp et al. (2013)
*Coprinopsis cinerea*	Multi-functional cellulase gene mfc	Growth substrate, highest activities of endo-β-1,4-glucanase (0.48 U/mL), total cellulase (0.26 U/mL), and endo-β-1,4-xylanase (38.10 U/mL) in culture liquid Cabm44, uses a 275 bp gpd fragment from *A. bisporus*	Yang et al. (2011)
Cricket *Teleogryllus emma*	Novel endogenous β-1,4-endoglucanase (EG) gene belonging to the glycosyl hydrolase family 9 (GHF 9)	Optimal pH and temperature of pH 5.0 and 40 °C. *K*_m_ and *V*_max_ values for digestion of carboxymethyl cellulose 5.4 mg/mL and 3118.4 U/mg	Kim et al. (2008)

## Application of consolidated bioprocess (CBP) with engineered host

Presently, commercial cellulosic plants for ethanol use individual hydrolysis and fermentation or SSF bioconversion techniques (Lynd et al. 2017). Although a Consolidated Bioprocess (CBP) configuration is defined as the combination of all production and secretion of the saccharolytic enzyme, polysaccharides hydrolysis, and available sugars fermentation within a single system is predicted to enhance process economics. The engineering of hosts like *Saccharomyces cerevisiae* is one of the favored methods for the development of CBP organism in which its ability to utilize cellulose was improved by expressing heterologous cellulase encoding genes (den Haan et al. 2015). The *S. cerevisiae* strain has been reported for effective fermentation of glucose obtained from pretreated LCB (Fujita et al. 2004; Yarbrough et al. 2015). Although no literature is found on the engineering of an *S. cerevisiae* strain, with the background of a natural strain isolate, having partial cellulolytic abilities which can help in the glucose fermentation obtained from pretreated biomass, which is a desired property for a CBP process. Some genetically different strains were transformed to produce core fungal cellulases, i.e., endoglucanase (EGII), β-glucosidase (BGLI), and cellobiohydrolase (CBHI) in different combinations and expression configurations for the identification of a suitable genetic background for effective cellulolytic secretion. In the analysis of activity levels of the secreted enzyme, the copy number of a gene, substrate specificities, along with the transformants hydrolysis and fermentation yield and the bioconversion efficiency of the partially cellulolytic yeast transformants for the pretreated corn cob and corn husk. This study showed a higher secretion titer by cellulolytic strains with the YI13 genetic background, and 1.34-fold higher concentrations of glucose (g/L) were obtained with cellulolytic transformants compared to the control in which equal amounts of each enzyme type was present. The co-production of BGLI and EGII from the 1:15 secretion ratio (unit of cellulase activity per gram of dry cell weight) resulted in the conversion of 56.5% of the cellulose present in corn cob into glucose in hydrolysis procedure and yielded 4.05 g/L ethanol. This study concluded that choosing an optimal genetic background and secretion ratio of cellulase activity can enhance the production of cellulosic ethanol by consolidated bioprocessing yeast strains (Davison et al. 2019b).

## Cutting-edge bioinformatics approach via molecular docking

A computational tool known as molecular docking for protein–ligand interactions study and the structure prediction of the intermolecular complex formed between the molecules. It is a critical method that places ligand (a small molecule) in the binding sites of the receptor (macromolecular target) for determining the binding. In molecular docking, the binding mode is called the best match depending on the structures of proteins found between the ligand and lowest energy scoring pose determined by an energy scoring function (Ezat et al. 2014; Huang and Zou 2010; Sharma and Jha 2010).

β-Glucosidase completes the bioconversion process by the hydrolysis of residual cellobiose or cellotetraose into glucose. Cellobiose, an intermediate product, also strongly inhibits the endoglucanase and exoglucanase activity and is considered a key hurdle in enzymatic hydrolysis. Therefore, for the prevention of this inhibition process, the removal of the cellobiose unit is required and thus became a very crucial step in understanding the β-glucosidase catalytic activity for enzyme efficiency improvement, subsequently helping in the formation of an enhanced β-glucosidases. Less information is found related to the catalytic interactions between β-glucosidase and cellobiose. Considering this, an attempt was made to understand the *Acinetobacter* sp. cellulase binding efficiency that four polysaccharides subunits, cellobiose, cellotetraose, cellotetriose, and laminaribiose, while using the molecular docking method (Selvam et al. 2017).

## Innovative bioprocess development: synergistic enzymes applications

Large quantity requirements of economic enzymes due to an increased number of biotechnological applications have subsequently increased the use of lignocellulosic feedstock as a potential energy source (Branco et al. 2010). Numerous techniques have been reported for the development of highly efficient cellulase (Ike et al. 2010), such as the use of UV-irradiated mutants (Ike et al. 2010) along with macro- and micro-mixing on enzymatic hydrolysis of lignocellulosic substrates into fermentable sugars (Chakraborty and Gaikwad 2010) and cellulase gene overexpression (Ribeiro et al. 2010), etc. Although lignocellulose degradation efficiency is not up to the mark due to the presence of both hemicellulose and cellulose, it is available as a carbohydrate source in plant material. As they are linked alternatively to each other, and action of individual xylanase and cellulase can only hydrolyze this holocellulose partially. Considering this, an approach for a simultaneous expression of both cellulase and xylanase is reported, in which a multi-functional cellulase gene from *Ampullaria crossean* stomach in Guangdong Province of South China (Cheng et al. 2009), showed that the exo-β-1,4-glucanase, endo-β-1,4-glucanase and endo-β-1,4-xylanase activities (Li et al. 2004; Wang et al. 2003). This gene can develop a novel engineering strain for the production of both cellulase and xylanase. During over expression of cellulase gene, over-glycosylation occurs causing low protein product expression level. The MFC, a multi-functional cellulase gene expressed in *Coprinopsis cinereae*, a basidiomycete large fungus that has the potential of exogenous genes overexpression (Cheng et al. 2009).

## Excessive solid concentrations utilization

The end-product inhibition can act as a limiting factor during the enzymatic hydrolysis of cellulosic feedstock-based biorefinery. Thus, excessive solids concentration can decrease water utilization and reduce equipment and distillation expenses during hydrolysis of cellulosic feedstock. This strategy can provide an effective approach for generating high concentrations of end-products of hydrolysis, such as cellobiose and glucose. It has been observed previously that the inhibition rate of cellulases end-product is limited for the hydrolysis process of lignocelluloses in high-solid conditions (André et al. 2003; Kristensen et al. 2009). Therefore, it can be assumed that relieving the inhibition of end-products has become the main challenge with this approach and also in enzyme engineering (Andrić et al. 2010).

## A critical factor of optimal enzyme load during various industrial applications

The cellulolytic organism’s genomes encode a huge series of catalytic subunits that are evolved to overcome the challenges that occurred due to the heterogeneity of the chemicals and complexity in the structures of the naturally available lignocellulosic substrates. Moreover, some fine-tuning is performed through the expression regulation of the cellulases genes to the ratio of each of the cellulases for getting the maximum hydrolysis (Kunitake and Kobayashi 2017). Some of the most significant factors that majorly influence the conversion of the cellulosic substrate from recombinant strains are a combination of the transcription machinery, genetic background, selection of the ideal cellulase gene along with the copy number of the gene (Li et al. 2017b). The adjustment of optimal concentration and ratios of all the single cellulases in a heterologous system is a challenging step to attain the more effective hydrolysis process utilizing while using lower enzyme dosages (Li et al. 2017b). This can improve cellulolytic enzymes efficiency and serve as an active research area in which efforts are made to understand the synergy displayed by the cellulases combinations and ratio optimization (Den Haan et al. 2013). The target of this study was mainly the reduction in enzyme load for effective cellulose hydrolysis and eventually reducing the enzyme cost. Hence, it is clear that the difference in cellulase secretion abilities of *S. cerevisiae* can be clarified in terms of several factors along with genetic background impact (Davison et al. 2016, 2019a; Marín-Navarro et al. 2011). Subsequently, internal and external pressures can affect the secreted recombinant protein yield in *S. cerevisiae* by using a background stress-tolerant strain, which can substantially affect the possibility and effectiveness of the bioethanol production using cellulosic materials (Gasser et al. 2008). The study of a total of thirty (30) natural isolates of *S. cerevisiae* was reported for the enhanced secretion activity along with other industrially important properties that are required for the lignocellulosic ethanol production process (Davison et al. 2016). A strain YI13 having a high secretory phenotype suggested 3.7 [Cel7A (CBHI)] and 3.5 [Cel5A (EGII)] fold higher secreted enzyme activity over control laboratory strain. Other industrially important properties were also observed in YI13, e.g., high titer of ethanol, growth vigor, high temperatures tolerance (37 °C and 40 °C), ethanol (10% w/v), along with several concentrations of an inhibitory compounds cocktail that are normally present in lignocellulose hydrolysate.

## Improvement in enzymes purification technique with enhanced specificity

Purification is the second most crucial step in the fermentation process due to the involvement of a total 60% cost of the whole process. The downstream process (DSP) of cellulase production involves series of conventional purification methods, e.g., ion exchange and gel filtration chromatography followed by precipitation and membrane filtration individually or in combination with associated limitations (Sadhu et al. 2013b; Singh et al. 2015). Although several effective purification techniques have been reported at the laboratory scale, there are still some drawbacks associated with them. For example, the chromatographic technique is complex in large-scale applications as it require costly chemicals and loss of protein yield (Hofman and Thonart 2001). The risk of particulate plugging can degrade the purification efficiency of the enzyme in the case of membrane-based filtration. Similarly, ammonium sulphate low selectivity toward target proteins can be seen in the ammonium sulphate precipitation technique (Bajaj et al. 2009). An aqueous biphasic system (ABS) is an alternative to these conventional methods to overcome the drawbacks. It is a liquid–liquid extraction process that offers a suitable environment for various biomolecules recovery and a cost-effective method. It decreases the production cost by decreasing the number of steps in the downstream process. ABS has been identified as a fruitful method for biological materials recovery and is frequently utilized to extract and purify enzymes, cell organelles, proteins, and nucleic acids (Raja et al. 2011). Therefore, ABS can be served as an easy step for scaling up at industrial level production of biomolecules (Goja et al. 2013).

The use of a basic polymer polyethylene glycol (PEG) and sodium citrate salt ABS was reported to extract extracellular cellulase from *B. subtilis*. The physicochemical interaction between cellulase and phase forming chemicals determines the partitioning behavior of cellulases in the system depending on the differences in their hydrophobicity, volume extension, electrochemical charges, and surface properties (Mazzola et al. 2008). Recent purification techniques utilized for the purification of cellulases are listed in Table 3.

**Table 3:** Exploration of recent purification method for enhanced cellulase activity

Strain and cellulase	Purification methods	S.A (IU)	Yield (%)	Purification fold	Mol. Wt. (kDa)	References
*Bacillus tequilensis* G9	Ammonium sulfate precipitation (70%)	26,505 ± 90	21.7	3.6	43	Dar et al. (2019)
	Acetone precipitation	44,602 ± 50	21	3.74		
	Ion-exchange chromatography technique (DEAE)-cellulose column (25 × 2 cm) equilibrated with 20 mM PBS (pH 7.4)			9.89		
*Pleurotus ostreatus*	Ammonium sulphate precipitation	36.8	54	10.5	58	Okereke et al. (2017)
	Dialysis	52.0	30.5	14.9		
	Gel filtration Chromatography	61.0	17.4	17.4		
*Bacillus subtilis*	Polyethylene-glycol (PEG)/sodium citrate aqueous biphasic system (ABS)	–	82.86	4.8	32–46	Ho et al. (2017)
*Bacillus* sp. H1666	Acetone precipitation followed by ion exchange and size exclusion chromatography		29	66.23	40	Harshvardhan et al. (2013)
*Rhizopus stolonifer var. reflexus* TP-02	Ammonium sulphate followed by dialysis, ultrafiltration, and Sephadex G-100	121.31		34.96	40–46	Tang et al. (2012)

## Reusability of cellulolytic enzymes: a recent advancement in cellulase immobilization

Tremendous research has been carried out over the past decades to fulfill the high demand for cellulases in various industrial processes and strategies to enhance the production of cellulases. In addition, also novel microorganisms were employed. It was found that native cellulases had low stability and activity problems along with high cost. These problems could be reduced in two ways. First, the production of cellulase must be economically feasible or by immobilization, which can provide better stability and applicability of the enzyme for the industrial processes (Cao 2005). Immobilization can improve the activity and performance of specific cellulase by reducing the amount of enzyme required for the process (Cao 2006). For the hydrolysis of different cellulase immobilizing techniques have tried which involved chitosan covalent immobilization (Su et al. 2012), on water-soluble copolymer (Liang and Cao 2012), in hybrid matrices by sol–gel encapsulation (Ungurean et al. 2013), on magneto responsive graphene nano-supports (Gokhale et al. 2013), etc. Most of the recent cellulase immobilization techniques and their outputs are listed in Table 4.

**Table 4:** Different immobilization techniques for cellulase nanoparticles and their characteristics

Immobilization approaches	Characteristics	References
Styrene/maleic anhydride copolymer nanoparticles	Improved stability against pH changes	Wang et al. (2018)
Phyto-silver nanoparticles synthesized using *Oxalis stricta* plant leaf extract	Effective on extracellular fungal amylase and cellulase	Singh et al. (2019)
Chitosan–cellulase nanohybrid and immobilization on alginate beads	Hydrolysis of ionic liquid pretreated sugarcane bagasse	Saha et al. (2019)
Multi-layered magnetic hollow particles	Rapid magnetic responsivity, high, bio-activity adsorption ability, and easily separated via magnet from a solution mixture	Raza et al. (2019)
Magnetic gold mesoporous silica nanoparticles core shell	Improvement of enzymatic activity and thermal stability	Poorakbar et al. (2018)
Reusable magnetic combi-CLEA cross-linked enzyme aggregate	Shows remarkable increase in the half-life of all three enzymes, bioethanol concentration increases to 1.82-fold as compared to free enzyme	Perwez et al. (2019)
Co-immobilized magnetic nanobiocatalyst: simultaneous immobilization of Pectinex® and Celluclast® amino-functionalized magnetic nanoparticle (MNPs): cross-linking by glutaraldehyde	Antioxidant extraction from waste fruit peels	Nadar and Rathod (2019)
Cellulase 32 immobilized magnetic nanoparticles (cellulase@MNPs)	Biomass hydrolysis by immobilized cellulase with 425 sonications found more effective than without sonication	Ladole et al. (2017)
Chitosan-coated iron oxide nanoparticles with APTES conjugated cellulase	Most effective for polyphenol release and the transformation of glycosidic to aglycosidic form of quercetin	Kumar et al. (2019)
Fe3O4-NH2@4-arm-PEG-NH2, a novel magnetic four-arm polymer–nanoparticle composite	Showed wide pH and temperature ranges, high operational stability, and good storage stability	Han et al. (2018)
Magnetic and silica nanoparticles	Improve catalytic efficiency of *Trichoderma reesei* cellulase for enhanced saccharification	Grewal et al. (2017)
pH-responsive lignin-based magnetic nanoparticles	Cellulase recycling and application of industrial lignin and increase the extra value	Dong et al. (2019)
Iron-tolerant *Pseudomonas stutzeri* biosynthesized magnetic nanoparticles	Photo-catalytically active and increased thermal stability	Desai and Pawar (2020)
Amino-functionalized magnetic nanoparticles	An activity-tunable biocatalyst for extraction of lipids from microalgae	Chen et al. (2018b)
Polymethacrylate particles (ICP) as the biocarrier grafted with ethylenediamine (EDA) and glutaraldehyde (GA)	for the hydrolysis of carboxymethyl cellulose	Chan et al. (2019)
Polyvinyl alcohol/Fe_2_O_3_ magnetic nanoparticles	Degrade microcrystalline cellulose	Liao et al. (2010)
Fe_3_O_4_ NPs	Magnetically recoverable biocatalyst for the decomposition of corncob	Zhang et al. (2016)
MnO_2_ NPs	Hydrolysis of agricultural waste for bioethanol production	Cherian et al. (2015)
Covalent immobilization on a uniform, monodisperse polyurea microspheres	Improve catalytic activity and stability	Sui et al. (2019)
MgO–Fe_3_O_4_ linked cellulase enzyme complex	Improves the hydrolysis of cellulose from *Chlorella* sp. CYB2	Velmurugan and Incharoensakdi (2017)
Fe_3_O_4_ nanoparticles with copper as ligand	Biocatalytic applications	Abbaszadeh and Hejazi (2019)
Biobased magnetic hollow particles (BMHPs) with glutaraldehyde via Schiff base reaction to produce multi-layered magnetic hollow particles (MMHPs)	Pens up a new strategy to immobilize enzymes, and the created MMHPs constitute a promising platform for immobilizing enzymes and other bio-macromolecules	Raza et al. (2019)
Carboxymethylcellulose sodium salt (CMC) and Cu_2_O nanoparticles	Nanocomposites exhibit photocatalytic activity to the Methyl Orange oxidation in the presence of H_2_O_2_ and air oxidation	Spiridonov et al. (2020)
Bleached eucalyptus fibers as cellulose source and cobalt ferrite nanoparticles (CoFe_2_O_4_)	Exist a positive linear relationship between the magnetic properties and the loading degree of the fibers	Pineda et al. (2020)
Cellulose–polyaniline–silver nanoparticles composites	To produce self-supported films constituted of polyaniline and silver nanoparticles	Oliveira et al. (2018a)
Carboxymethyl cellulose stabilized cobalt nanoparticles (CMC-Co) catalyst	The effects of catalyst amount, a combination of the two pollutants in a solution, and repeatability tests were also performed and results were discussed	Kamal et al. (2019)

### Physical adsorption

This process works on the concept of reversible immobilization in which physical adsorption or attachment of enzymes occurred onto the support material through weak non-specific forces such as hydrogen bonds, Van der Waals, and hydrophobic interactions (Kumar 2009). This process has high commercial potential as its processing cost is comparatively low and straightforward. But it has drawbacks like leakage of immobilized enzyme reduces the activity of enzymes after multiple cycles (Won et al. 2005). There are three modes from which enzymes and bio-carrier usually interact, i.e., hydrogen, electrical bonding, and hydrophobic (Nussinovitch 2010; Won et al. 2005). Biocarrier screening is an essential step towards the improvement of an effective bio-catalytic process for specific industrial applications. The selection of solid support can be done by three fundamental measures, i.e., physical/mechanical strength, cost-effectiveness, and chemical stability (Guisan 2006). Affinity binding is also one of the physical adsorption methods that involve selecting biomolecules to be applied in the immobilization of enzymes.

### Covalent bonding

This process comprises a multipoint linkage between the support surface and enzyme, which causes strong attachment. Support reuse is not permitted in the process because there are possibilities of breakage in the enzyme support during the elution of enzymes. Also, the substantial reduction in the activity of enzyme after multipoint covalent attachment is possible because of the enzyme’s movement restriction and conformational changes on they might be due to restriction of the movement of the enzyme, conformational changes on the carrier surface, or mass transfer effects (Singh et al. 2013). The effective enzyme separation was reported with Fe_3_O_4_ magnetic particles through magnetic field modulation. Although agglomeration occurred, affecting the mass transfer rate (Lima et al. 2017).

### Cross-linking immobilization

An attempt was made on cellulase immobilization without a carrier as no solid carrier is required to obtained high enzyme activity per unit volume (Seow and Yang 2017). Cross-linked enzyme aggregates were reported to be an effective alternative to the traditional solid carriers immobilization methods due to high volumetric activity, stability, economic production, easy process, reusable, no use of no purity enzyme, and no solid carrier requirement (Sheldon 2011b; Yamaguchi et al. 2018). No mechanical resistance (improvable by dehydration hardening and intense cross-linking) (Garcia-Galan et al. 2011), enzyme mobility reduction, and limitations in the internal mass transfer are the drawbacks of cross-linked enzyme aggregates which can be problematic for macromolecular substrates (Cui and Jia 2015).

Native enzymes’ less activity was believed due to carbamates inactivation on the enzyme surface with lysine residues and local pH reduction due to carbonic acid formation (Dijkstra et al. 2007). Improvement in the enzymatic activity by supercritical carbon dioxide was reported by various scientists (De Souza Melchiors et al. 2017; Melgosa et al. 2015). The cross linked enzyme aggregate (CLEA's) is a type of immobilization without application of any carrier. In CLEAs, cellulase is cross-linked with glutaraldehyde for obtaining active and stable enzyme preparation (Podrepšek et al. 2019). An immobilized cellulase on amino-functionalized MNPs (Fe_3_O_4_@mSiO_2_-NH_2_) using glutaraldehyde as a cross-linking agent by Schiff base formation showed efficient prevention in enzyme leaking and improvement in enzyme activity (Fang et al. 2016).

## The industrial perspective of cellulolytic enzymes

Attention toward cellulase and other cellulolytic enzymes has considerably increased due to their importance in the hydrolysis of cellulosic materials to fermentable sugars to produce value-added products such as biofuel (Soares-Silva 2016; Kumar and Verma 2020b). Some recent developments in the applications of cellulases are described below. Some recent developments in the applications of cellulases are described below.

## Paper and pulp industries

Considering the limited availability of petroleum resources (Sepehri et al. 2020), cellulosic materials have attracted much more attention as they have renewable and biodegradable features (Shi et al. 2019; Thomas et al. 2018). Pulp dissolution with more than 90% of pure cellulose was found in the special commercial pulp with many downstream applications, e.g., micro filters, cellulose esters/ethers, viscose rayon, and lyocell (Arnoul-Jarriault et al. 2015; Li et al. 2016b). For all these applications, viscose rayon production utilized nearly three-quarters of the dissolving pulp production (global annual production was ∼ 8 million tons) (Kumar and Christopher 2017). In the production of dissolving pulp mainly two commercial processes are involved, i.e., acid sulfite (AS) and pre-hydrolysis kraft (PHK). However, AS is preferred more because of the compatibility with different raw materials along with the alkali recovery units effectiveness (Li et al. 2016b). PHK has shown a low reactivity problem because accessible hydroxyl groups are not synthesized by the alkaline peeling reaction of PHK pulping. Hence, further upgrading technique is much needed to increase the reactivity of PHK pulp that can determine the consumption of toxic carbon disulfide in the xanthation stage of production of rayon. Various studies have been reported for further PHK pulp up-gradation, e.g., mechanical treatment (Tian et al. 2014), enzyme treatment (Li et al. 2018a), chemical treatment (Wang et al. 2014), microwave (Liu et al. 2018), ionic liquor, and electron beam irradiation (Chen et al. 2017). From all these tested methods, cellulase treatment was found as an effective approach with green and mild behavior. Cellulase treatment may increase the reactivity of fock in dissolving pulp from 47.7 to 79.9% with 2 μ/g and 2 h cellulase dosage (Miao et al. 2014) and observed that cellulase treatment could increase fock reactivity of dissolving pulp. A new cellulase spraying method is also reported for upgrading the dissolving pulp with an increase in fock reactivity from 51.2 to 82.6% under 60% pulp consistency condition with 1.5 mg/g and 12 h cellulase dosage (Li et al. 2018a).

Although the efficiency of cellulase was hindered due to the inherently compact nature of the cell wall structure and aggregation of fibrils, that was more complicated after multiple bleaching and pulping stages of the PHK production process. Large amounts of lignin and hemicellulose removals can create spaces and pores for new aggregation and generate structures with less accessibility (Engström et al. 2006). The cationic polyacrylamide (CPAM) addition can induce the cellulase adsorption ratio by the bridging and patching effect, eventually increasing the efficiency of cellulolytic enzymes. Apart from the integrated mechanical treatment, cellulase treatment can serve as an alternative practical method for upgrading the dissolving pulp. Mechanical refining has been used in several areas (Mou et al. 2014), e.g., a commercial process of papermaking (Chen et al. 2012), production of nano-/micro-fibrillate fiber (He et al. 2018), and digestibility of enzymes (Wang et al. 2012). The remarkable increase in breaking length, stretch, and tensile index (Chen et al. 2012) 41.6%, 33.5%, and 30.8%, respectively, was observed from a beaten pulp of 54°SR as compared to an unbeaten pulp. Only a few studies have been reported to date describing the effect of depth refining on hydroxyl groups’ liberation and accessibility of enzymes, mainly while the activation of dissolving pulp using cellulase. In terms of considering reactivity, a critical factor of dissolving pulp can determine the consumption of toxic chemicals such as carbon disulfide in rayon production. A detailed refining to improve the PHK pulp before the treatment of cellulase was studied for describing that the mechanical refining can not only enhance the reactivity by liberating additional hydroxyl groups, but also increases the efficiency of cellulases by improving the accessibility of enzymes. This study showed a clear increase in fock reactivity of refined pulp at beating degree of 50° SR from 54.8% of the original at beaten degree 19° SR to 78.0%, mainly due to the changes in the intermolecular hydrogen bond.

Additionally, an increase in a range of 39.7–71.2%, adsorption ratio of cellulase from the refined pulp (30–50° SR) was reported to verify the enzymatic accessibility improvement. An integrated process involving mechanical refining and 0.5 mg/g of cellulase dosage treatment resulted in better efficiency than the control with 1 mg/g pulp of cellulase dosage considering the increase in reactivity and reduced viscosity. Other pulp properties, e.g., length of the fiber, the value of water retention, specific surface area, morphology, and crystallinity, showed the depth refining positive effect on dissolving pulp activation during treatment cellulase (Wang et al. 2020b).

The ideal concept for biorefinery is the complete utilization of LCB (Van Heiningen 2006). A well-known commercial practice for dissolving pulp is kraft-based Pre23 hydrolysis that supports this ideal concept as they separate hemicellulose, cellulose, and lignin in several streams (Miao et al. 2014). The dissolved pulp can be used for several applications, e.g., cellulose derivatives and viscose rayon production. The adjustment of reactivity and viscosity is one of the most critical challenges in dissolving pulp production. Cellulase treatment has been reported as an effective approach for reducing viscosity and increasing enzyme reactivity. The use of endoglucanase in the treatment of cellulase mainly degrades amorphous cellulose present on the surface of the fiber and in-between the microfibrils that enhance the exposure of crystalline surface, pulp reactivity, and swelling ability (Ibarra et al. 2010).

The recycling of cellulases can be an effective method for reducing the cost of enzymes. After the enzymatic treatment of dissolving pulp, cellulase as a catalyst remains in the liquor phase itself. Reuse and recovery of the liquor can reduce the enzyme cost and attain zero-water release in the stage of enzymatic treatment. Cellulase recycling has been studied previously in the year 1971 in the lignocellulose bioconversion process. The recyclability of various cellulases can be obtained considering the enzymatic substrate during the study of biofuel production. Economic point is an important concern during the enzymatic treatment of dissolving pulp for industrial-level application. The cellulase recycling strategy using fresh cellulase was reported to activate the dissolving pulp, i.e., enhancing fock reactivity and decreasing viscosity. According to the study, 48.8–35.1% of cellulase activity can be recovered in five recycle rounds from the filtered liquor that can be utilized again for the dissolving pulp enzymatic treatment. The recycling of cellulase and additional fresh 1 mg/g led cellulase to the pulp with 470 mL/g viscosity and 80% fock reactivity comparable with 2 mg/g cellulase charge (Wang et al. 2016).

## Food and feed applications

### Clarification of juice

Cellulolytic enzymes are useful for food processing industries such as juice clarification. The disruption of the vegetable cell wall is caused during pressing and releasing the internal juice in the grape juice processing. The freshly pressed juice is cloudy and turbid due to the colloidal dispersion of pectin and is considered a significant hurdle in juice processing (Irshad et al. 2017; Tapre and Jain 2014). In addition to this, cellulose and hemicellulose-like polysaccharides get settled during the storage resulting in a flavorless and clear-colored water-like juice is a significant problem (Vaillant et al. 2001). The commercial enzyme preparations mainly using hemicellulases, cellulases and pectinases are widely used in fruit juice industries to overcome these problems. In their soluble forms, these enzyme preparations play a vital role in the hydrolysis of such compounds, improve filtration, juice clarification, and stabilization (Romero-Cascales et al. 2012; Sandri et al. 2011).

Immobilization methodology has appeared as an effective approach to develop heterogeneous biocatalysts and enhance enzyme recovery (Krajewska 2004). Enzyme immobilization has shown stability in difficult reaction conditions, simplifies the separation of the enzyme from the reaction systems, allows biocatalyst reusability during the continuous process and mainly during multiple reaction cycles, and increases their commercial, industrial importance (Garcia-Galan et al. 2011). Previous studies have suggested that the enzyme immobilization could be utilized for the apple, carrot, grape, pineapple, and orange juices clarification (Sojitra et al. 2017). Although there is no immobilization method was industrially applied for such purpose due to costly processes and economically less feasible. Thus, this opened a new area for new research. Generally, covalent immobilization has been preferred as it may prevent desorption of enzymes from the support during the reaction (Garcia-Galan et al. 2011; Rodrigues et al. 2013; Royvaran et al. 2016). Additionally, support type is a critical factor in the enzyme immobilization process, as it may affect the enzyme loading amount and its operational stability and catalytic activity (Cao et al. 2003; Miletić et al. 2009; Pal and Khanum 2011).

Usually, small size biocatalysts are gaining more attraction due to their high specific surface area available for the attachment of enzymes. Although the separation step from the reaction medium may be difficult due to the sedimentation of insoluble components in juice clarification. Immobilized enzymes separation from the sediments is difficult by conventional processes, e.g., centrifugation and filtration. Thus, magnetic particles could be utilized as efficient support for the immobilization of enzymes to reduce these limitations because their magnetic core offers efficient, easy, and quick enzyme separation from the reaction mixture by utilizing the external magnetic field; additionally, its size can be personalized to offer high surface and increased enzyme activity (Ansari and Husain 2012; Laurent et al. 2008) since these magnetic particles are susceptible to oxidative and acidic conditions. Hence, their functionalization is very important for stability maintenance (Wu et al. 2008).

Carrier-free biocatalysts are found to be another approach for enzyme immobilization. Cross-linked enzyme aggregates (CLEAs), generally prepared by the direct cross-linking of different enzyme preparations (Sheldon 2011a), offer several advantages for increased and concentrated enzyme activity with high stability and economical production process because of the elimination of an additional carrier (Cao et al. 2003). Therefore, two different magnetic biocatalysts were prepared for the application in juice clarification targeting to help biocatalyst separation (Dal Magro et al. 2018).

### Cellulase in animal feed improvement

There are several other advantages associated with cellulase-producing bacteria, such as probiotic activity. (Kewcharoen and Srisapoome 2019) The probiotic bacteria are rich in cellulases and are vital to the utilization, digestion, and absorption of animal feed, mainly in young animals with imperfect digestive systems (Li et al. 2020). Also, cellulases from bacteria such as *Bacillus subtilis* are used for the nutritional enhancement of animal feed. These processed feeds are exploited to enhance body weight gain, milk yield, etc.

Further, animal feed processing with these enzymes can help remove anti-nutritional factors present in grains and other cellulosic materials (Jayasekara and Ratnayake 2019). Tahir et al. (2005) suggested that the processing of corn–soybean meal of broiler chicken via a combination of cellulase and hemicellulases has a synergistic effect on the performance of the feed. Lucio et al. (2021) presented a comprehensive review on the effect of enzymes as zootechnical additives in animal feed. The review provided evidence on the positive effect of cellulase addition in food supplement processing for animals.

## Cellulase applications in biorefinery industries

Environmental pollution-related concerns and limitations in oil supply with continuous increase in global demand for energy have substantially induced the urgent requirement of cost-effective alternatives towards the renewable, clean, and maintainable production of biofuel (Hyeon et al. 2016). Hence, various products of biorefinery industries such as bio-butanol, biomethane, bioethanol, biohydrogen, and biodiesel have been introduced as an effective alternative to the petroleum-based resources to bio-based and eco-friendly energy resources (Abraham et al. 2014; Alexandri et al. 2019). Concerning the biorefinery industry, lignocellulosic plant biomass is the most effective, economic, renewable, and most abundant natural resource that has been widely studied without affecting or competing with the food industry (Zabed et al. 2016). The proper bioconversion process of lignocellulose obtained from the plant biomass require the involvement of enzymatic hydrolysis, where cellulolytic enzymes play a key role in breaking down the cellulosic polysaccharide structure into fermentable sugar molecules (Lynd et al. 2008; Obeng et al. 2017; Thapa et al. 2020). Enzymatic hydrolysis using cellulases offers a more efficient bioconversion system due to having more specificity towards substrates, better saccharification, less complexity, and environment friendly when compared to non-enzymatic hydrolysis system (Ungurean et al. 2013).

A study reported that effective polymer–enzyme bioconjugates with more stability and activity for practically using cellulases in LCB hydrolysis. A dual cross-linking (DC) approach was reported for the synthesis of a novel 3D network of CMC grafted copolymers of 2-acrylamido-2methyl propane sulfonate and acrylamide [CMC-g-poly (AMPS-co-AAm)] hydrogels. Graphene oxide (GO) nanosheets were used as nano-filler and physical cross-linker, which were forming H-bonds between polymeric chains for the preparation of GO@CMC-g-poly (AMPS-co-AAm) networks. For examining the effect of GO content on the effectivity of synthesized architectures in conjugation to a control enzyme known as PersiCel1. Immobilization of PersiCel1 on the GO reinforced hydrogels which showed a remarkable retaining approx. 60% of its maximum activity at 90 °C and increased specific activity and storage stability. Compared to free control PersiCel1, the immobilized enzyme resulted in a 154.8% increase in alkaline treated sugar beet pulp bioconversion, whereas the PersiCel1/neat-Hydrogel showed 66.7% increment, under the same conditions (Ariaeenejad et al. 2020).

*Gracilibacillus* SK1, a halophile isolated from Yuncheng Salt Lake was reported for cellulolytic activity, in which production of cellulase was strongly affected by the culture medium salinity with the highest level in the presence of 10% NaCl. A multi-component enzyme system was observed during the substrate specificity test with a combined activity of β-glucosidase endoglucanase and exoglucanase, which is confirmed by zymogram analysis. The crude cellulase with high activity and stability over broad temperature range, i.e., 40–70 °C, pH ranging from 6.0–10.0 and 7.5–17.5% NaCl concentration with the temperature and pH optima of 60 °C and 8.0, respectively, with 12.5% NaCl, resulted in thermo-alkali stable and halo-stable properties. Also, it showed high stability in the presence of hydrophobic organic solvents.

Corn stover and rice straw saccharification were reported using cellulase with 0.678 and 0.502 g yields of reducing sugars with g/L of dry substrate, respectively, that was later utilized to produce ethanol using *Saccharomyces cerevisiae*. The crude cellulase from *Gracilibacillus* sp. SK1 reported the efficiency of bioethanol production with the yield of ethanol 0.186 g g/L of the dry substrate and a 52.8% conversion percentage (Yu and Li 2015). The extraction of natural components from plants using enzymes has been widely studied. They offer many advantages compared to traditional methods, e.g., simple operation, more efficient, and economic process. Many studies in the biomolecule extraction process are mainly reported using cellulase, pectinase, and glucosidase for the hydrolysis and degradation of cell wall components and subsequently releasing the intracellular ingredients. The conventional enzyme-based extraction is complex, tedious, and needs high-energy consumption, eventually limiting its large-scale application (Chen et al. 2016). Anionic liquid-based enzyme extraction system of geniposide, coupled with in situ hydrolysis from *Eucommia ulmoides* Olive barks, is reported using enzymatic hydrolysis. A single cellulase was used to extract and hydrolyze geniposide in a continuous system (Chen et al. 2018a).

*Aspergillus fumigatus* Z5, with the lignocellulosic decomposing ability and has the capacity to produce cellulase, was reported under SsF using lignocellulosic feedstock as substrates. The optimization of cellulase production from *A. fumigatus* Z5 and resulted that carboxymethyl cellulase (CMCase) and filter paper enzyme (FPase) production at 50 °C, 80% initial moisture, initial pH 4.0, and 7% initial inoculum with CMCase and FPase activity, 526.3 and 144.6 U/g dry weight, respectively. *A. fumigatus* Z5 reported a total of eight types of cellulases under zymogram analysis in the presence of cellulose-containing materials in the culture. The crude cellulase from *A. fumigatus* Z5 was found capable of hydrolyzing pretreated corn stover for bioethanol production by using *Saccharomyces cerevisiae*, with 0.112 g g/L dry substrate ethanol yield (Liu et al. 2011). The study aimed to produce a cellulase blend and evaluate its application in a SSF process for second-generation ethanol production from sugar cane bagasse.

The residual solid fraction obtained from a diluted acid and alkaline pretreated sugar cane bagasse was subjected to cellulase production and ethanol fermentation using a SSF process. A bioreactor with two inoculum concentrations (5 and 10% v/v) was used for cellulase production by inoculating a larger inoculum size for high yield. Tangential ultrafiltration in the hollow fiber membrane was used to concentrate the cellulase extract, which was stable for 300 h at 37 °C and 50 °C. This on-site cellulase blend was used to produce bioethanol by using the PDC fed-batch SSF process. The feeding method avoided the characteristic problems of limitations in diffusion by decreasing the high solid presence:liquid ratio at any time and resulted in a high ethanol concentration at the process end (100 g/L) and showed a 78% fermentation efficiency resulting from the ratio of 380 L of ethanol per ton of sugar cane bagasse PDC (Maeda et al. 2013).

A filamentous fungi *Penicillium oxalicum* for cellulase production in submerged fermentation in shaking condition was reported with a cellulase activity of 0.7 FPU/mL. After the process optimization, cellulase production was increased by 1.7-fold resulted in a maximum cellulase activity of 1.2 FPU/mL in 8 days of incubation. A successful scaled-up system of 7-L fermenter was studied for cellulase production under controlled conditions, which showed a decrease in incubation time from 8 to 4 days for attaining a similar cellulase yield. pH and temperature optima for crude cellulase activity were pH 5 and 50 °C, respectively. The crude cellulase retained approximately 50% and 26% of its activity at 48 h and 72 h, respectively. *P. oxalicum* hydrolytic efficiency was comparable to commercial cellulase preparations and indicated its potential for lignocellulose hydrolysis application (Saini et al. 2015).

*Aspergillus fumigatus* isolated from chemically polluted microhabitats was reported for cellulase production with a maximum cellulase activity at 30% (v/v) ionic liquids (ILs). Variations in the initial cellulase activity were observed in each IL, a longer half-life in most ILs than in buffer, with high conformational stability of the enzyme essential for sustaining the residual activity inappropriate media. Remarkably, 1–3 M NaCl can help in cellulase activation. A compatible IL-cellulase system based on the cellulase was reported with its use in significantly improving the rice straw saccharification rate from 53 to 88% as compared to the control, representing its effectiveness for the competent transformation of lignocellulose to glucose in a single-step process (Xu et al. 2014).

## Commercial cellulase availability and eminent market suppliers

Cellulase has widespread applications in industries such as the paper and pulp industry, textile, food, and biorefinery. Some of the cellulase-producing companies on commercial scales are Novozymes (Denmark), Genencor-Danisco (Rochester, USA), DuPont (USA), Worthington Biochemical Corporation (Lakewood, USA), Dyadic (Jupiter, USA), Quest Intl. (Sarasota, Florida, USA), BASF (Germany), Kerry Group (Ireland), DSM (Netherlands), Chr. Hansen (Denmark), Rhom-AB enzymes (Rajamaki, Finland), Amano Enzyme Inc. (Nagoya, Aichi, Japan), Zhongbei Bio-Chem Industry Co., Ltd, (China) and Advanced Enzymes (India) (Markets and Markets 2020; Verardi et al. 2012). Some commercially available cellulases with their source and critical properties are tabulated in Table 5.

**Table 5:** List of commercial cellulase producer and suppliers with its source and characteristics. Source: Verardi et al. (2012), and company’s catalogue

Commercial/market name	Company/supplier	Location/country	Source	Characteristics
Accellerase® 1500	Genencor-Danisco	Rochester, USA	Recombinant *Trichoderma reesei*	Endoglucanase: 2200–2800 U/g β-Glucosidase: 450–775 pNPG U/g Best operational stability at temperature: 50–65 °C and pH: 4.0–5.0
	DuPont Industrial Biosciences	USA	Recombinant *Trichoderma reesei*	Cellulase: 82 mg/mL Xylanase: 51 mg/mL
Bio-feed beta L	Novozymes	Bagsvaerd, Denmark	*T. longibrachiatum* *T. reesei*	FPU: < 5 U/mL Cellobiases: 12 U/mL
Biocellulase A	Quest Intl	Sarasota, Florida, USA	*A. niger*	β-Glucosidase: 32 Um/L High stability at pH 5 and temperature 55 °C
Cellubrix (Celluclast)	Novozymes	Bagsvaerd, Denmark	*T. longibrachiatum* *A. niger*	FPU: 56 U/mL Cellobiases: 136 U/mL
Cellic CTec2 (cellulase, enzyme blend)	Sigma Aldrich	Missouri, United States	*Trichoderma reesei* *Aspergillus niger*	Variable efficiency
Cellulase 2000L	Rodhia-Danisco	Vinay, France	*T. longibrachiatum* *T. reesei*	FPU: 10 U/mL
Cellulyve 50L	Lyven	Colombelles France	*T. longibrachiatum* *T. reesei*	FPU: 24 U/mL
Cellulase AP 30 K	Amano Enzyme Inc	Nagoya, Aichi, Japan	*A. niger*	β-Glucosidase: 60 U/mL High stability at pH 4.5 and temperature 60 °C
Cellulase NLT 10,000 U/g	Zhongbei Bio-Chem Industry Co., Ltd	China	*Trichoderma *spp.	Cellulase activity: 10,000 U/g Optimum activity at pH 4.5 and temperature 40–50 °C
Energex L	Novozymes	Bagsvaerd, Denmark	*T. longibrachiatum* *T. reesei*	FPU: < 5 U/mL Cellobiases: 19 U/mL
GC220	Genencor-Danisco	Rochester, USA	*T. longibrachiatum* *T. reesei*	FPU: 116 U/mL Cellobiases: 215 U/mL
GC440	Genencor-Danisco	Rochester, USA	*T. longibrachiatum* *T. reesei*	FPU: < 5 U/mL Cellobiases: 70 U/mL
GC880	Genencor-Danisco	Rochester, USA	*T. longibrachiatum* *T. reesei*	FPU: < 5 U/mL Cellobiases: 86 U/mL
Novozymes 188	Novozymes	Bagsvaerd, Denmark	*A. niger*	FPU: < 5 U/mL Cellobiases: 1116 U/mL
Rohament CL	Rhom-AB Enzymes	Rajamäki, Finland	*T. longibrachiatum* *T. reesei*	FPU: 51 U/mL Cellobiases: 28 U/mL
Spezyme CP	Genencor-Danisco	Rochester, USA	*T. longibrachiatum* *T. reesei*	FPU: 49 U/mL
Ultraflo L	Novozymes	Bagsvaerd, Denmark	*T. longibrachiatum* *T. reesei*	FPU: < 5 U/mL Cellobiases: 20 U/mL
Viscozyme L	Novozymes	Bagsvaerd, Denmark	*T. longibrachiatum* *T. reesei*	FPU: < 5 U/mL Cellobiases: 23 U/mL
Viscostar 150L	Dyadic	Jupiter, USA	*T. longibrachiatum* *T. reesei*	FPU: 33 U/mL Cellobiases: 111 U/mL

The global cellulase market is spread worldwide, with a major focus in the Asia Pacific, Europe, the Middle East, and Africa, Latin America, and North America (TMR 2021). The increasing production and application of biofuel in North American and Latin American countries have projected them to be the leader in the market in the coming years (TMR 2021). Also, Asia Pacific regions with fast-growing economies like India and China can be viewed as a lucrative opportunity and expected to signal substantial market growth. The shift in focus from petrochemical-based refineries to bio-based biorefinery with greener technologies has increased application enzymes. These enzymes are lignocellulolytic, pectinolytic, amylolytic, and chitinolytic enzymes with the major market contribution of the cellulolytic enzyme (Kumar and Verma 2020a). This increase in demand is pushing researchers, academicians, government agencies, and industries to improve the yield and efficacy of cellulases.

## Techno-economic aspects of the cellulase production on large scale

**Fig. 4:**
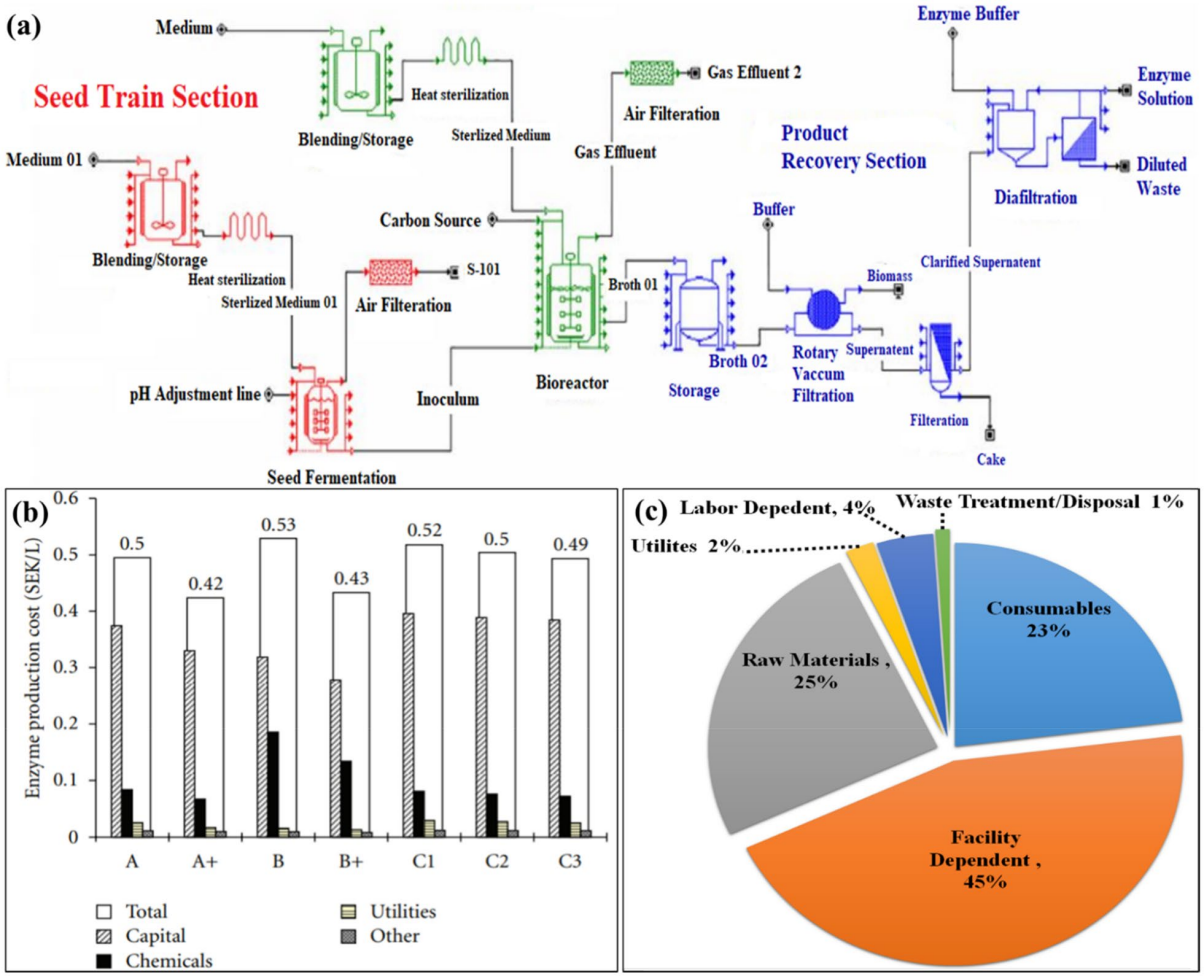
Techno-economic aspect of cellulase production: **a** generic flowsheet of a cellulase production process with filamentous fungi [adapted with permission (Ferreira et al. 2021)]. **b** Cost contributors of enzyme production in (Swedish kronor) SEK/L ethanol. A, A+: *pretreated liquid fraction*, B, B+: *pretreated liquid fraction and molasses* and C1, C2, C3: pretreated liquid fraction and pressed pretreated slurry with a total WIS content of 1%, 2% and 3%; +: 1.5-fold specific activity (adapted from Barta et al. 2010). **c** Composition of the enzyme cost for recombinant β-glucosidase production using the baseline scenario (adapted and modified from Ferreira et al. 2018)

The cellulase applications in several industries, as discussed above and the challenge to retain the cost of enzyme production is an important parameter. Another critical factor is in controlling the economics of cellulase or enzyme reusability. Thus, a focus on developing the on-site production of cellulase using cheap lignocellulosic substrates has been suggested. Barta et al. (2010) studied the process design and economics of softwood-based ethanol plants, emphasizing on-site cellulase production utilizing different carbon sources. The study showed that in ethanol production at an industrial scale, the capital cost (0.42–0.53 SEK/L ethanol) is the main contributor, where 60–78% of the cost comes from enzyme production. Also, the study suggested that introduction of enzyme production step may cause a decrease in overall ethanol yield by 5–10 L/tonne, which was 270 L/dry tonne of raw materials when the commercial enzyme was used. Further, the application of pretreatment before the use of biomass to cellulase production is suggested for enhanced cellulase production in a short incubation time. Barta et al. (2010) also demonstrated that pretreated liquid fraction supplemented with molasses could be an economical alternative in producing ethanol at a lower cost (4.71 and 4.82 SEK/L) (Fig. 4b).

Ferreira et al. (2021) analyzed the production cost of lignocellulose degrading enzymes and reveals that the most preferred organism and methods for lignocellulolytic enzyme productions are *Trichoderma reesei* (Teleomorph *Hypocrea jecorina*) and filamentous fungi using submerged and SsF, respectively (Fig. 4a). The techno-economic parameters evaluations suggested wide variation in cost on several process designs where the choice of raw materials/substrates and capital-related costs are generally the motor element of the enzyme production cost (Fig. 4c). The employment of low-cost medium utilizing cheaper and abundant raw materials such as agricultural and forestry residues can decrease the overall cost of enzyme production. Also, the production of auxiliary enzymes enhances the pretreatments and saccharification efficiency. The study suggested that other important parameters that are key to the economics of cellulase production are production titer, yield, and volumetric productivity.

The fungal sources of cellulase enzymes are used commercially, and supplementing the fungal enzymes with their lacking enzymatic activities can help in enhanced hydrolysis yield and subsequently help achieve low-cost hydrolysis (Ferreira et al. 2018). Ferreira et al. (2018) performed the techno-economic analysis of integrating β-glucosidase in a recombinant *E. coli* to industrialize a low-cost enzyme for their application in biorefinery (Fig. 4c). The major observations were that facility-dependent costs contribute significantly to the plant and recombinant enzyme production in *E. coli* is high compared to conventional methods. However, the cost of the enzyme could be reduced by using cheaper substrates, improve volumetric productivity and inoculation process apart from using a cheaper induction strategy. The *E. coli* strain is easy to handle at an industrial scale due to optimized upstream and streamlined downstream processes *that* help control the overall cost to produce the enzyme.

One of the limiting factors during cellulase-based enzymatic saccharification of lignocellulosic is unproductive adsorption of cellulases on lignin causing the reduction in enzyme activity (Gao et al. 2014; Siqueira et al. 2017). The addition of additives, e.g., non-ionic surfactant (tween) (Mukasekuru et al. 2018), polymer, e.g., polyethylene glycol (Arias et al. 2017), and non-catalytic protein, e.g., bovine serum albumin and soybean (Brondi et al. 2019, 2020) can help in overcoming the lignin mediated enzyme reduction and further results in enhanced enzyme activity at low enzyme load with high solid loading. The low cost and global abundance of soybean protein make it stand out lignin-blocking additive (Klein-Marcuschamer et al. 2012; USDA 2021). Brondi et al. (2020) performed a retro-techno-economic analysis of the application of soybean protein as an additive during saccharification in an integrated (1G-2G) biorefinery. The study suggested that experimentally increasing biomass conversion (up to 80%) and reducing enzyme loading to 5.6 FPU/g with soybean additive resulted in an economically integrated biorefinery.

Zhuang et al. (2007) demonstrated an economic assessment of the methodology, i.e., SsF and submerged fermentation (SmF) used for the production of cellulase and its subsequent application in the conversion of fibrous biomass to bioethanol. The economic analysis suggested that the unit cost of cellulase production was around $15.67 and $40.36 per kilogram for the SSC and SmF, respectively. The deflation and inflation in the prices were calculated based on a factor of 0.9 and 1.1. Monte Carlo simulation-based sensitivity analysis suggested the unit cost of production using SSC is lower than SmF with high certainty of 99.6%. Thus, suggesting SSC as a suitable low-cost in-house cellulase production methodology, thereby causing a reduction in overall ethanol production cost (Zhuang et al. 2007). The techno-economic survey of cellulosic biorefineries is recommended to adapt on-site co-production of lignocellulolytic enzymes using cheap agro-residues substrates (De Souza et al. 2021; Olofsson et al. 2015). Thus, this decrease in enzyme cost is expected to overcome the limitations of costly enzymes during saccharification in integrated biorefineries.

## Conclusion and future prospects

Microbial cellulases obtained from LCB feedstock as a substrate is an efficient approach and can meet a high level of industrial demand. However, further studies are required to scale up cellulase production for the improved utilization of the strain at the industrial level. Future perspectives of the study may involve identifying new strains with the potential of multi-enzyme production and further developing pretreatment methods for effective utilization of lignocellulosic feedstocks. Identifying new genes from potential microorganisms for performing the genetic manipulations leads to improved catalytic property of cellulases. In addition, the development of inexpensive purification methodology at a large scale and molecular characterization of immobilized enzymes to increase its reusability and further kinetic parameter analysis of an immobilized enzyme can be an added advantage towards the industrialization of the bioconversion process. Also, as the enzyme production cost is key to its designated industrial application thus studies suggested that on-site co-production of multiple enzymes with emphasis on utilizing engineered strains and controlling process parameters are feasible methods for controlling the overall cost of the industry.

## Data Availability

Not applicable.
